# Pathos & Ethos: Emotions and Willingness to Pay for Tobacco Products

**DOI:** 10.1371/journal.pone.0139542

**Published:** 2015-10-20

**Authors:** Francesco Bogliacino, Cristiano Codagnone, Giuseppe Alessandro Veltri, Amitav Chakravarti, Pietro Ortoleva, George Gaskell, Andriy Ivchenko, Francisco Lupiáñez-Villanueva, Francesco Mureddu, Caroline Rudisill

**Affiliations:** 1 Universidad Nacional de Colombia, Bogotá, Colombia; 2 Università degli Studi di Milano, Milano, Italy; 3 University of Leicester, Leicester, United Kingdom; 4 London School of Economics and Political Science, London, United Kingdom; 5 University of California Riverside, Riverside, California, United States of America; 6 Columbia University, New York City, United States of America; 7 Universitat Pompeu Fabra, Barcelona, Spain; 8 Universitat Oberta de Catalunya, Barcelona, Spain; 9 CRENoS—Centre for North South Economic Research, Cagliari, Italy; University of L'Aquila, ITALY

## Abstract

In this article we use data from a multi-country Randomized Control Trial study on the effect of anti-tobacco pictorial warnings on an individual’s emotions and behavior. By exploiting the exogenous variations of images as an instrument, we are able to identify the effect of emotional responses. We use a range of outcome variables, from cognitive (risk perception and depth of processing) to behavioural (willingness to buy and willingness to pay). Our findings suggest that the odds of buying a tobacco product can be reduced by 80% if the negative affect elicited by the images increases by one standard deviation. More importantly from a public policy perspective, not all emotions behave alike, as eliciting shame, anger, or distress proves more effective in reducing smoking than fear and disgust.

**JEL Classification:**

C26, C99, D03, I18

**PsycINFO classification:**

2360; 3920

## Introduction

### 1.1 Motivation

The typical model of decision making used in economics postulates that choices are made following the consequentialist criterion: agents take decisions by rationally evaluating the consequences of a given action [1]. In this framework, the only emotions considered are those experienced as the consequences of a particular action. Much less attention has been devoted to the role of emotions experienced at the time of decision-making. The emotions experienced at the time of decision making, however, may have a complex and profound effect on choice, for at least three reasons. First, since decisions are context-dependent, a decision maker’s emotional state may act as a circumstantial factor that distorts choice in comparison to another situation where the decision maker’s affective state is neutral. Second, since consequences are often evaluated with regards to a reference point, emotions matter as they may shift this reference point. Finally, since certain moods can affect the way in which the decision maker perceives the future, the affective states emerging at the moment of choice may change the beliefs that the decision maker has about what consequences to expect. In particular, emotions can either change the prospective gains and losses with respect to the reference point itself, or make the decision maker discover his/her preferences by realizing the possible emotional states that he/she will experience when the consequences materialize [2].

The literature on behavioural economics has explored the three dimensions above, but has not tackled in sufficient depth two important issues: the specific and possibly differential role of strong emotions, and the behavioural consequences of different types of emotions. Regarding the first issue, typically, strong emotions such as fear and disgust have not been investigated in behavioural economics on account of the difficulty of manipulating such emotions in a laboratory setting. Regarding the second issue, the psychological literature ([3]; [4]) amply warns us that the relations between cognition, emotion, and behavior may vary significantly across different affective stimuli. For example, different affective states are known to differentially impact cognition, with negative affect (*negative valence*) said to impact information processing by increasing attentional focus [5], and the intensity (*arousal*) of the affective state said to affect the depth of processing [6]. Similarly [7] have shown that while positive affect leads to the use of broader categories, negative affect leads decision makers to employ narrower categories in their subsequent decision making.

The consequences of this variation in affective states for choice and behavior have rarely been explored in experimental settings in behavioural economics. This is especially true for strong emotions, albeit there is a nascent literature in game theory that assesses the impact of mild emotions, like the effect of fairness in strategic interactions [8][9]. One notable exception is [10], which use experimental primes related with fear and anxiety to proxy violence in Afghanistan. However, their interest is purely in violence and do not focus on the differences across emotional states. The psychological and neuroscience literature has been much more involved with emotions and also strong emotions, especially in the health domain ([11]; [12]); nevertheless, the experimental study of the relationship between emotion and behavior has been relatively neglected.

Using the results of a unique large scale and multi-country study—where negative versus positive emotions, intensity of exposition, and intensity across different emotions are all recorded—we contribute to this emerging but still nascent body of research in behavioural economics by looking at the effect of strong emotions on choices.

More specifically, in this article we investigate the impact on cognition and behavior of a different and more granular range of emotions (including strong ones) elicited through exposure to pictorial warnings describing the health and social consequences of tobacco consumption. We identify the relationships between various emotions and our dependent variables through instrumental variables. The instruments are the graphic stimuli, which are administered through a randomization algorithm. Since our instrumentation is based on random exogenous variations, we can interpret as causal the estimated effect of emotions on the outcome variables. We use a novel database from a study on Tobacco labeling options conducted simultaneously in 10 European countries. The database is based on a study conducted by the authors on behalf of the EC (European Commission). The experimental conditions are 84 combined warnings (i.e., combined text and image warnings; CW hereafter) on the health and social consequences of smoking that are expected to generate different types of emotional responses, together with a set of text-only warning (control conditions, TW hereafter). Combining behavioural (in terms of elicited willingness to buy and pay for a tobacco product) and psychometric scales of responses we attempt to isolate the effects of different emotions. We document the following findings. First, the most basic emotions we examined in our study were successful in significantly reducing decision makers’ action propensity and thoughts of smoking. Specifically, our findings suggest that the odds of (i.e., the intention of) buying a tobacco product can be reduced by 80% if the valence (the negative versus positive nature of the emotion) of the emotions elicited by the CWs increases by one standard deviation. Additionally, the impact of a standard deviation variation of different emotional scales on intentions to quit smoking (for smokers), avoid smoking (for non-smokers), risk perceptions, and other cognitive variables changes by a minimum of 50% of a standard deviation to up to 110% of a standard deviation. Second, we also find that not all strong emotions behave alike. When the images elicited emotions like shame, anger, anxiety, and distress they were much more successful in reducing the decision maker’s odds of buying a tobacco product (by about 82%) than when the images elicited emotions like fear and disgust (by about 66%).

### 1.2 Related Literature

The first systematic review of the role of emotions in economic theory was done by [13], at a time when a program of research specifically addressing this topic was yet to be established. Developments in research in behavioural economics led to the emergence of such a program. First, existing experimental evidence on violation of asset integration and reference dependence suggests that reference effects are important components of choice, and that *expected* or *counterfactual emotions* can play an important role in shaping the reference point ([2]; [14]; [15]; [16]). By reference point we mean a particular choice option with which all other alternative are compared and with respect to which other alternatives are re-framed in terms of gains and losses. Expected emotions are a decision maker’s expectations of how (s)he would feel once the outcome of the decision has materialized. Such emotions are not experienced at the time of choice. Thus, at the time of choice, expected emotions are merely cognitions about future affect.

Besides its impact via expected or counterfactual emotions, the relevance of emotions in decision-making might also be explained by two other mechanisms. Both these mechanisms are associated with immediate affect (as opposed to future affect). (On the distinction between immediate affect and expected emotions and on the different approaches of economists—mostly focusing on the latter—and psychologists—mostly focusing on the former—please see [17]) That is, the emotions that are experienced during the course of a decision or a choice. One such mechanism is through *integral emotions*. Integral emotions are those emotions that are normatively related to the judgment or decision at hand. For example, an integral emotion could be the typically strong emotions (i.e., experienced at a high level of arousal) that a decision maker experiences at the time of choice while thinking about the potentially bad or good consequences of her choice. Such integral emotions are very different from expected emotions and play the role of unveiling one’s own preferences (thus departing from the conventional assumptions that preferences are well defined, as stressed by [2]). As pointed out by [14], expected emotions are fundamentally cognition of future utilities, while anticipatory emotions are emotions of future utilities. The second mechanism is through *incidental emotions* that arise from contingencies that are normatively unrelated to the decision or task at hand [2]. These emotions could arise from a past event (e.g., an unrelated event that made the decision maker happy or angry earlier in the day), the decision maker’s physiological states (e.g., being hungry), or both.

Different strands of literature in the psychological domain have documented that emotions may play a role on preferences and action without activating cognitive processing, or activating it *post actum*, or that emotions play the role of mediator between cognition and action. First, these contributions suggest that by limiting itself to expected emotions (which are always and necessarily the result of cognition) conventional economic models are omitting an important driver of decision-making. One such literature is the “mere exposure effect” paradigm ([18], [19]). These experiments keep cognition constant (e.g., via recall of images) and show how preference formation (e.g., “liking” of an image) is positively associated with multiple exposures to the same treatment. The results from this paradigm suggest that a relationship between affect and preferences can indeed occur without conscious cognitive processing ([20]; [21]; [18], [19]). Sometimes cognition is actually prevented by cultural characteristics: [13] stresses how the research on cross-cultural differences highlights the fact that some cultures do not have a cognitive label for some emotions. The lack of a cognitive label would suggest that emotions can exist in a cognitive void. Second, it is interesting to note that cognition in many situations is actually an *ex post* reaction, one that is constructed to justify a choice that was largely determined by affect, as in the case of cognitive dissonance [22]. Yet another literature that attests to the complex and non-linear relationship between cognition and emotional processes is the established consensus in neuroscience that has led to the formulation of the “somatic marker hypothesis” ([23], [24]). This hypothesis considers emotion at the core of the human decision making process and argues that the absence of emotion can lead to suboptimal decisions even when cognitive faculties are left intact. These studies used patients with damages that deprived their brains of particular emotional signals. In the decision tasks given to them the patients relied on a reasoned cost-benefit analysis of numerous and often conflicting options involving both immediate and future consequences. Such brain impairment degraded the speed of their deliberations (e.g., choosing between two items took patients a very long time because of endless reasoned analyses of the pros and cons of each option), and also degraded the decision quality (i.e., patients made suboptimal choices). More precisely, Damasio and colleagues do not argue that emotion is always beneficial. They concluded that it is beneficial to decision-making when it is integral to the task, but it can be disruptive when it is unrelated to it. This strand of literature suggests that postulating a causal mechanism where only cognitively processed emotions matter is empirically weak, since sometimes emotions come after cognition to pave the way for a course of action.

Experimental and behavioural economics provide further evidence that emotions can play a strong and constructive role in decisions. For example, consider some of the evidence against the standard assumption of maximization of individual gains, such as those demonstrated in the Ultimatum Game or Dictator Game. These seeming anomalies can easily be accounted for by emotions like feelings of fairness or unfairness [25]. A similar emotion-based account could be constructed to explain the empirical evidence that minimal environmental elements (odors, sights, sounds) can drive choices, which are significantly different from those performed in a more aseptic setting ([2]; [14]). Finally, the strong evidence of projection bias in behavioural choices [26] also suggests that decisions and choices made for the future are fundamentally impacted by emotions. Human subjects seem to be affected by a cognitive bias related to the role of the emotions (*hot-cold empathy gap*): they attribute to non-visceral factors what is indeed due to the affective state in which they are deciding. In other words they are incapable of correctly predicting their future state, when they will not be affected by the current emotion, and thus tend to project their current state to the future in an inaccurate manner. As a result emotions affect intentions to act in the future or inter-temporal decisions with respect to a neutral affective state. Yet, existing research in behavioural economics has focused only on milder emotions and does not address strong basic emotions and the different effects of different kinds of emotions.

To some extent the same can be said about the new behavioural approach to policy making that goes under the label of “nudge”. The idea of behavioural “nudges” is highly relevant to the domain of health [27]. This approach, in fact, concerns classical nudges mostly focusing on heuristics and biases unrelated to strong emotions. (For instance, one notable example is the ‘save tomorrow’ success story and the literature on the financial implication of dynamic inconsistency ([28]; [29]; [30] Hence, by focusing on CWs as policy measures aiming to dissuade tobacco consumption via the use of strong imagery, in this article we show that strong emotions can also be at the core of the behavioural approach to effective policy. This also points to the fact that perhaps the concept of policy “nudges” is in need of further refinement as we find that strong emotions also appear to serve as effective nudges in the domain of health.

In the specific domain of tobacco consumption studies there is already a vast literature that tries to assess the impact of pictorial warnings on knowledge, salience, cognitive processing and the intention to quit/avoid smoking (as shown by the systematic reviews performed in [31]; [32]). The diffusion of CWs is usually justified on the basis of limited implementation cost by government with respect to mass media campaigns and their reach among smokers [33]. This popularity has contributed to the design of large surveys assessing the effectiveness of CWs. Nevertheless, the number of studies that couple Randomized Control Trial (RCT) methodology with the elicitation of a behavioural choice is very limited (for exceptions see [34]; [35]).

The novelty of our study with respect to the tobacco consumption literature is twofold: first, we provide evidence for a representative population at the European level with robust RCT methodology; second, we explore in depth emotions and quantify their effect as a conduit to modifying behavior. The three advantages of our RCT as compared to simple surveys should be highlighted: (1) randomization algorithm allows detecting causal impacts rather than mere correlation; (2) we have measures of behaviours (and not cognitions alone); and (3) we have a very detailed and granular measurement of emotions that goes beyond the traditional focus on the “fear effect” alone.

We can summarize the three key contributions of this article as follows: (1) more generally, we investigate the role of strong emotions in decision making, thus contributing to the literature in behavioural economics; (2) we add to the existing evidence on the effectiveness of pictorial warnings in dissuading tobacco consumption by employing a large scale, multi-country data set and a more robust experimental design and methodology using instrumental variables to infer causal impacts; and (3) we also contribute to the ongoing scientific and policy debate on the behavioural foundations of policy.

### 1.3 Research Design, Methods and Main Results

The data for this study comes from a project financed by the European Commission. The aim was to test the effectiveness of 84 new health warnings made up of a combination of text and pictures (Combined Warnings, CWs) to be used on packages of tobacco products. Each of the 14 text warnings had 6 images associated with it, with each image aimed at providing a pictorial representation of the risk described in the text warning. For instance, for the textual warning ‘smoking causes 9 out of 10 lung cancers’ there were six different pictures that vividly and factually conveyed the health consequences of this risk (please see examples in Fig A and Fig B in Section E of S1 File). Similarly, there were 7 other text messages that focused on the health risks of smoking with pictures vividly conveying the physical consequences of smoking, often showing the adversely affected organs. Besides these 8 textual warnings, there were 6 other textual warnings that focused on the health consequences of smoking, but each textual warning was matched by symbolic or evocative images, instead of gruesome organ-based representations. Hence, there were two types of CW, which we term as ‘Organs’ (8 textual warnings each matched by 6 pictures) and ‘Social’ (6 textual warnings each matched by 6 pictures). Therefore, for each of the 14 textual warnings we tested 6 pictures with the aim to select the three most effective CWs (42 CW in total). More precisely, the aim of the study was to test the 84 CWs in order to select the 42 best performers (3 out of 6 per text message), which will appear on packages of cigarettes, RYO and waterpipe tobacco starting in May 2016 on the basis of the new Tobacco Products Directive 2014/40/EU. Directive 2014/40/EU requires the top of the front and the back of the package to host a CW, covering 65% of the display area.

This comprehensive study was based on RCT methodology, and was conducted on a representative (of the 18–65 year old population) sample of 8000 European citizens, covering 10 Member States of the European Union. To the best of our knowledge, this is the largest RCT multi-country study ever conducted on tobacco pictorial warnings, and the first one at the EU level. The Experimental flow can be found in Section D of S1 File; the full experimental protocol can be found in Section C of S1 File.

In this work we are interested in analyzing the consequences of emotional responses on behavioral measures. However, we cannot just perform a simple regression analysis to conclude that emotions affect behavior, as we may have problems of endogeneity: there could be an omitted variable that is correlated with both sets of measures—generating a correlation even though emotions may have no effect on behavior; or the causal relationship may be the opposite—behavior may cause emotions. Overall, a simple regression analysis is unable to address these issues. One typical solution is to use an instrumental variable: a variable that is uncorrelated with the error term of the dependent variable (behavior), but that has an impact on the dependent variable through the explanatory variable (emotions).

Our identification strategy is indeed based on instrumental variables. The instruments are the stimuli applied in the study, that is, the textual and pictorial warnings administered to the participants. We use them as an instrument to study the relationship between our explanatory variable of interest—the emotions—and our dependent variable of interest—behavior (intentions to quit smoking, willingness to pay for cigarettes, and so on). There are two requirements to be met for the instrument to be valid. The first one is the exogeneity of the instrument with regards to the dependent variable, that is, the instrument must be administered independently of the dependent variable of interest avoiding measurement error, omitted variables, and reverse causation/simultaneity. Because the different CWs in the experiment were administered randomly, using a RCT, we have a fully exogenous variation.

Second, we need the instrument—the CWs—to be correlated with the (endogenous) explanatory variable—emotions—conditioned on the other explanatory variables. We have both a theoretical and an empirical justification of the instrument. Theoretically, these images are clearly directed towards the activation of emotions as motivators of action (i.e., the *affect heuristic* described in [36]). There is a vast literature on the emotional effects of pictures ([37]; [38]; [39]), which tests images from the International Affective Picture System (IAPS containing both Mild and Strong images) and recovers emotional reactions using the same SAM and PANAS scale we used. According to this literature there is a very high correspondence between the judgments of emotions reported using SAM, PANAS, and physiological measures of emotions such as skin-conductance, eye-blinks, facial corrugator muscles, etc. (i.e., [39]). This suggests that emotional measures are less cognitively mediated than other behavioural measures such as willingness to buy. This further supports our identification strategy based on instrumenting the emotional response and then identifying the effect of emotions on behavior.

Empirically, we report in the Section A of S1 File, a set of Tables (Tables A-H in Section A of S1 File) where we show the correlation between the entire set of stimuli and the emotional measures. As can be seen from the Tables, the instrumentation is robust.

As a result we have a valid instrument to study the impact of variations of emotions on behavior. Regressing behavioural choices on instrumented emotional responses allows us to quantify the effects of different emotions on behavior.

The study included both self-reported and behavioural choice, in the form of Willingness to Pay (WTP) elicitation. In addition to the effect on behavior, we estimate the effect of emotions on two different cognitive variables: attitudes towards the health risks of smoking and the intention to talk and discuss the consequences of tobacco consumption with other people (a word-of-mouth variable which we interpret as a proxy for depth of processing since, after all, only in-depth processing of the message would motivate the individual to talk to other people and attempt to persuade others against smoking). Moreover, we also look at the effect on the intention to quit and avoid smoking. We measure risk perceptions because a number of studies indicate that emotions may have a direct impact on risk perception [14]. For example it has been shown that emotions induce agents to buy insurance after an earthquake, or after having known someone who was assisted during floods or earthquakes, even without any change in informational content about risk [40]. We measure depth of processing because researchers like [6] have shown that different emotions activate differential depths of processing.

The rationale for the use of behavioral intentional (*conative*) indicator is the possible presence of projection bias: under a “hot” state, it is likely that subjects will overestimate their intention in the future [41]. If this activation occurs, it is more likely that participants will report a significant intention to quit/avoid smoking.

We find that even a small change in emotional valence (from positive to negative emotional state) reduces the likelihood of buying a cigarette pack and the willingness to pay for cigarettes in a significant way. We also find that both cognitive processing and the intention to act are affected. Similar effects are found in the case of emotional arousal. Investigating the effect of variations in basic emotions (the simplest emotional states, such as fear, disgust, etc., on which more complex emotions are built) shows a large array of effects on cognitive, conative, and behavioral variables. Finally, we also find that not all strong emotions are alike. When the images elicited emotions like shame, anger, anxiety, and distress they were much more successful in reducing the decision maker’s odds of buying a tobacco product than when the images elicited emotions like fear and disgust.

The remainder of the manuscript is organized as follows: Section 2 presents the experimental design and methodology, Section 3 the results, Section 4 discusses the robustness of the results, Section 5 concludes with a discussion of the implications of our findings.

## Materials and Methods

### 2.1 Experimental Design

As explained in Section 1, to address the relationship between emotions and behavior we use the data of a project commissioned by the European Commission to select appropriate CWs in terms of effectiveness on a number of response variables.

The experiment was conducted online in ten European countries with 800 respondents per country. The countries were selected to cover different regions (representing also the main cultural-institutional blocks) of the European Union, as well as to represent the different types of tobacco legislations that were present across Europe. At the time the experiment took place pictorial warnings on cigarettes packs were already in use (in the chronological order of introduction) in Belgium (2007), Romania (2008), United Kingdom (2008), France (2011), Spain (2011), and Denmark (2012), covering half our sample of respondents. They include Belgium, France, Germany (Continental bloc), Italy and Spain (Mediterranean bloc), Denmark and Sweden (Scandinavian bloc), United Kingdom (Anglo Saxon bloc), and Poland and Romania (Eastern bloc). In each country, the sample is representative of the 18–65 population bracket and is stratified by gender and age group (18–30, 31–50, 51–65). Participation was remunerated through a fixed fee. The experimental work was conducted from the 9^th^ of November 2012 to the 5^th^ of December 2012. The ethical authorization has been provided by The London School of Economics Research Ethics Committee. The latter gave the ethical approval for all the countries where the data were collected. The participants in the online panel are contacted by email and were asked to give their informed consent by clicking on an "Accept" or a "Reject" button (without formal acceptance the questionnaire does not start). The privacy policy and use of data is illustrated at the following link (http://www.cint.com/panelist-privacy-policy/), which is available on the webpage where the respondent was asked to give consent. Informed consent regarding privacy policy and use of data is listed under point 13 of The London School of Economics Research Ethics approval requirements. The Committee approved the study and as such also the procedure to require informed consent.

We consider textual warnings ((1) “Smoking causes 9 out of 10 lung cancers”; (2) “Smoking causes mouth and throat cancer”; (3) “Smoking damages your lungs”; (4) “Smoking causes heart attacks”; (5) “Smoking causes strokes and disability”; (6) “Smoking clogs your arteries”; (7) “Smoking increases the risk of blindness”; (8) “Smoking damages your teeth and gums”; (9) “Smoking can kill your unborn child”; (10) “Your smoke harms your children, family and friends”; (11) “Smokers’ children are more likely to start smoking”; (12) “Quit smoking—stay alive for those close to you” (13) “Smoking reduces fertility”; (14) “Smoking increases the risk of impotence”) and the six pictures associated with each of them. We are interested in testing the differential effect of plain information (text only) versus information integrated with imagery (CWs). Thus we have 14 control conditions (textual warnings) and 14 x 6 = 84 CW treatments. As indicated earlier, the CWs can be divided into two groups: 1) Organ-type combined warnings, with text and pictures related to effects of smoking on the body (48 CWs); 2) Social-type combined warnings, with text and pictures of a social nature (36 CWs).

Each respondent was subject to *seven* exposures, in three blocks (randomization was blocked by type of CWs in order to control interaction effects due to the nature of the stimuli one saw first. The literature shows that different types of pictorial warnings have different effects, [42]; [43]. More effective CWs might have produced a “flattening” of the response to the exposition of other types.): 1) one exposure to a textual warning alone; 2) three exposures to Organ-type combined warnings; and 3) three exposures to Social-type combined warnings. The order of the blocks was determined randomly, and within each block each CW was selected randomly from those of each type. The only restrictions were that each respondent saw each CW at most once, and saw at most one CW per text (i.e., they never saw two CWs with different pictures but with the same text). The randomization and counterbalancing algorithm used in the design of the online experiment ensured that we had enough statistical power to test the effectiveness of every CW, regardless of the fact that certain types are seen more than others.

For each exposure, the image (or text) was at first shown on the entire screen. Subsequently, as we elicited responses to various questions, the image (or text) was repeated on each screen in a smaller size. The questions comprised emotional, cognitive, intentional (or conative), and behavioural responses. After all the seven treatments were administered, the smokers were asked to answer a set of questions related to their smoking habits. All these questions are discussed in detail in the section below.

In Section D of S1 File we report the experimental flow, which includes additional explanations about the block randomization procedure. In Section E of S1 File we report some examples of the images (Fig A and Fig B, of Section E of S1 File) and in Section F of S1 File we report the number of observations per treatment.

### 2.2. Emotional, Cognitive, Conative, and Behavioural Measures

We included four sets of measures: emotional, cognitive, conative, and behavioural measures.

#### 2.2.1. Emotional Measures

**Fig 1:**
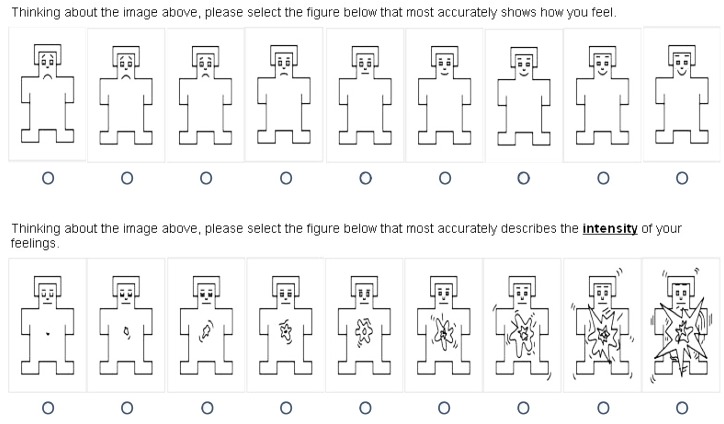
The SAM Scale

To record emotional responses we considered three different types of variables (elicited in the following order): (1) the Self Assessment Manikin (SAM), (2) Measures of fear and disgust; and (3) Four items of the Negative Affect Schedule. The SAM scale was originally developed by Lang (Lang, 1980), and later refined by many other researchers. In our study, we used the scale to ask respondents to indicate (a) the valence of the emotion (positive or negative), and (b) the level of arousal (intensity of the emotion) they experienced upon seeing each image. Both scales are represented below in Fig 1.

The fear and disgust measures were elicited via a 7-point scale, with the following questions: “Please indicate whether this image is frightening,” and “Please indicate whether this image is disgusting.” Both questions had identical anchors (1 = “Not At All” and 7 = “Very Much”).

In the literature on Tobacco consumption it is common to use the Positive and Negative Affect Schedule (PANAS hereafter; [44]). This corresponds to a set of positive and negative affect measures. In our study a reduced scale of five items were included, aimed at detecting distress, anxiety, anger, shame, and fear. The specific adjectives used for the corresponding scale on which the participants responded were "upset," "anxious," "nervous," "ashamed," and "afraid," respectively. In our final analysis we do not consider the last item (i.e., "afraid") as a question on fear was already administered prior to the PANAS scale. Responses to both questions are highly positively correlated (R = .7061 p-value = 0.0000, Spearman’s rho = 0.7073, p-value = 0.0000), and including one or the other measure makes no material difference to the pattern of results.

We adopted these emotional variables for the following reasons. In the psychology literature on emotions there have been various attempts to identify what is often referred to as *basic* emotions, on which all the other emotions are based. Our choice of the emotional response variables corresponds to the important types of basic emotions that this stream of research has revealed (e.g., [45]). In keeping with the criterion set by this literature for an emotion to be classified as basic, all emotions in our study (anger, fear, distress, disgust, anxiety, and shame) (a) are said to be hardwired (e.g., as defined by [46]; [47]), (b) relate to action tendencies or action propensities ([48]; [49]), (c) do not require propositional content [50], and (d) all but anxiety prompt dense neural firing [51].

In operationalization of these five emotions, the phrasing adopted was, “This scale consists of a number of words that describe different feelings and emotions. Read each word and then mark the appropriate answer next to that word. Indicate to what extent you feel this way in reaction to this image. Use the following scale to record your answers.” We included the adjectives Upset, Ashamed, Nervous, Afraid, and Anxious, presented every time in random order. All responses to these questions were on a scale anchored from 1 (very slightly or not at all) to 5 (extremely).

#### 2.2.2. Cognitive and Conative Measures

The cognitive measures used were: (a) risk perception and (b) depth of processing. The former was elicited through the following question: “Please indicate whether this image makes you more concerned about the health risks of smoking.” The depth of processing was elicited through the following question: “Please indicate on the scale below to what extent you think this image would encourage you to talk with other people about the dangers associated with smoking.” In both cases the answer was provided on a continuous scale anchored at 1 ("Not At All") and 7 ("Very Much").

The intentional (or conative) measures were differentiated for smokers and non-smokers. To the former we asked about their intention to quit; to the latter we asked their intention to avoid smoking. The specific question for the smokers was, “Please indicate on the scale below whether this image motivates you to quit smoking.” Non-smokers responded to the question, “Please indicate on the scale below whether this image motivates you to avoid smoking.” Again, we used scales that were anchored at 1 ("Not At All") and 7 ("Very Much").

#### 2.2.3. Behavioral Measures

The behavioral measure we used is the respondents' Willingness to Pay (WTP) for a pack of cigarettes. We adopted the following procedure. Subjects were shown a list of possible prices and were told to choose either one of them as the maximum price that they would be willing to pay for the packet of cigarettes, or to choose not to purchase it at any price. The exact question was phrased differently for smokers and non-smokers. The grid of prices that subjects saw was centered on the average price of a pack of cigarettes in their country of residence, and each point on the grid comprised a 6% variation in price. Half of the participants saw a grid that went from a minimum of minus 30% to a maximum of plus 30% of the average price in their country; the other half saw a grid that went from a maximum of plus 36% to a minimum of minus 30% of the average price in their country. The two types of pricing grids allow us to test if there is a difference in the stated WTP between the symmetric (i.e., -30% to +30%) and skewed (i.e., -36% to +30%) grids. This procedure is similar to a particular form of Multiple Price List procedure (e.g., [52]), in which participants are asked to select only the "switching point" [53]. Because these questions are not incentivized, and thus noisier [54] we believe our design is the easiest to understand (the question is formulated straightforwardly) and the single choice minimizes noisy response with respect to a list of multiple choices.

Our behavioural measure is not incentivized, both for budgetary and for licensing reasons (restrictions on selling cigarettes). The lack of incentives increases the noise [55], although it is well known from a large body of evidence that for this type of question it does not systematically alter the pattern of results ([54]; [1]).

Using these behavioural responses we construct two measures: (a) a dummy for the purchase decision (Willingness to Buy, WTB) depending on whether the respondent bought or chose not to buy, and (b) a Willingness to pay measure using the revealed prices (measured in terms of percentage variation with respect to the average price).

### 2.3 Identification Strategy

We created 98 dummy variables, corresponding to the 84 CW treatments plus the 14 control conditions (text only warnings). As we discussed in the Section 1.3 above, we use the CWs as an instrument of emotions, where the validity of the instrumentation is based on (1) the absence of correlation between the instruments and the error term of the dependent variable—cognitive and behavioural measures, which is ensured by the use of randomized assignment; and (2) a correlation between the explanatory variables—emotions—and the treatments—the CWs (which is due to the fact that CWs are deemed to generate emotional response and is corroborated by the results shown in Tables A-H of Section A of S1 File).

Following standard procedures for the use of instrumental variables, we first regress the endogenous independent variable (emotional responses) over the set of 98 treatments (excluding the constant): this gives us the predicted values. Then, in a second stage we regress the outcome measures (cognitive and behavioural variables) over the predicted value generated above, using Fixed Effect estimators to eliminate unobserved heterogeneity. For the case of the willingness to buy we report a two steps fixed effects logit model. Technically, one should directly run a maximum likelihood estimation, which is computationally much more expensive. In the end, since the two steps linear estimation (which is unbiased) provides the same results, we rely on the two steps procedure. This analysis generates our main results discussed below. Additional analysis, performed for robustness, is discussed in Section 4.

In Section B of S1 File, we report a set of Kruskal-Wallis tests in which we compare the education level, age, gender, smoking status, country, household size, marriage status, and occupation across treatments, to assess robustness of the randomization algorithm (Table I of Section B of S1 File). We report the test for each order of exposition, ensuring that the test assumption of independence of observations is ensured. The socio-demographic characteristics are balanced, suggesting that our randomization algorithm is robust.

## Results

In the Tables 1–8 below we report the results of the *instrumented* elicited emotions over the range of outcome variables (cognitive, conative, and behavioral) discussed earlier.

**Table 1:** Effect of Emotional valence.

	(1)	(2)	(3)	(4)	(5)	(6)
	WTP dummy	WTP	Intention to quit	Intention to avoid	Depth of processing	Risk perception
Valence	1.19 (.03)***	.01 (.00)***	-.86 (.01)***	-1.03 (.01)***	-.72 (.01)***	-.93 (.01)***
Const		-.10 (.00)***	6.54 (.06)***	8.09 (.05)***	6.46 (.03)***	7.45 (.03)***
N. Obs	15324	21768	20713	35207	55915	55920
LR test	1322.92***					
F test		141.39***	1958.11***	3912.07***	3994.12***	5931.75***
R2		.01	.05	.07	.03	.06
Hausman test	449.41***	73.39***	765.05***	1354.67***	1465.40***	2141.38***

Note: All the estimations are done with Two Stages Fixed Effects. Column (1) is a Fixed Effects Logit; columns (2)-(6) are Fixed Effects Linear regression. Each column corresponds to a regression; the dependent variable is indicated at the top of the column. Instruments are the 98 stimuli of the study (84 CWs and 14 textual warnings).

* indicates statistical significance at 10%

** at 5%

*** at 1%.

Standard errors in parenthesis. The Hausman test in all columns (including 1) is based on the linear estimator (Fixed Effects versus Two stages Fixed Effects).

**Table 2:** Effect of Emotional arousal.

	(1)	(2)	(3)	(4)	(5)	(6)
	WTB	WTP	Intention to quit	Intention to avoid	Depth of processing	Risk perception
Arousal	-.85 (.02)***	-.01 (.00)***	.63 (.01)***	.74 (.01)***	.52 (.00)***	.66 (.00)***
Const		.012 (.00)**	.284 (.07)***	.669 (.06)***	1.25 (.04)***	.78 (.04)***
N. Obs	15324	21768	20713	35207	55915	55920
LR test	1383.18***					
F test		150.73***	2171.26***	4122.68***	4268.36***	6126.08***
R2		.01	.06	.07	.04	.06
Hausman test	366.34***	78.11***	475.21***	765.43***	633.25***	1175.41***

Note: All the estimations are done with Two Stages Fixed Effects. Column (1) is a Fixed Effects Logit; columns (2)-(6) are Fixed Effects Linear regression. Each column corresponds to a regression; the dependent variable is indicated at the top of the column. Instruments are the 98 stimuli of the study (84 CWs and 14 textual warnings).

* indicates statistical significance at 10%

** at 5%

*** at 1%.

Standard errors in parenthesis. The Hausman test in all columns (including 1) is based on the linear estimator (Fixed Effects versus Two stages Fixed Effects).

**Table 3:** Effect of Distress.

	(1)	(2)	(3)	(4)	(5)	(6)
	WTP dummy	WTP	Intention to quit	Intention to avoid	Depth of processing	Risk perception
Upset	-1.78 (.05)***	-.02 (.001)***	1.23 (.02)***	1.46 (.02)***	1.08 (.01)***	1.29 (.01)***
Const		.00 (.00)	.26 (.07)***	.58 (.06)***	1.04 (.04)***	.75 (.05)***
N. Obs	15324	21768	20713	35207	55915	55920
LR test	1331.33***					
F test		117.01***	1884.72***	3590.88***	4186.99***	5249.69***
R2		.01	.06	.07	.04	.06
Hausman test	486.11***	110.57***	696.54***	1414.85***	1353.37***	2114.98***

Note: All the estimations are done with Two Stages Fixed Effects. Column (1) is a Fixed Effects Logit; columns (2)-(6) are Fixed Effects Linear regression. Each column corresponds to a regression; the dependent variable is indicated at the top of the column. Instruments are the 98 stimuli of the study (84 CWs and 14 textual warnings).

* indicates statistical significance at 10%

** at 5%

*** at 1%.

Standard errors in parenthesis. The Hausman test in all columns (including 1) is based on the linear estimator (Fixed Effects versus Two stages Fixed Effects).

**Table 4:** Effect of Shame.

	(1)	(2)	(3)	(4)	(5)	(6)
	WTB	WTP	Intention to quit	Intention to avoid	Depth of processing	Risk perception
Ashamed	-2.37 (.07)***	-.02 (.00)***	1.53 (.04)***	1.95 (.03)***	1.46 (.02)***	1.61 (.02)***
Const		.00 (.00)	.23 (.09)**	.26 (.08)***	.75 (.05)***	.72 (.06)***
N. Obs	15324	21768	20713	35207	55915	55920
LR test	1089.99***					
F test		98.69***	1365.67***	2858.01***	3542.03***	3711.57***
R2		.01	.04	.05	.03	.04
Hausman test	519.62***	60.03***	501.96***	1102.01***	1393.69***	1479.86***

Note: All the estimations are done with Two Stages Fixed Effects. Column (1) is a Fixed Effects Logit; columns (2)-(6) are Fixed Effects Linear regression. Each column corresponds to a regression; the dependent variable is indicated at the top of the column. Instruments are the 98 stimuli of the study (84 CWs and 14 textual warnings).

* indicates statistical significance at 10%

** at 5%

*** at 1%.

Standard errors in parenthesis. The Hausman test in all columns (including 1) is based on the linear estimator (Fixed Effects versus Two stages Fixed Effects).

**Table 5:** Effect of Anger.

	(1)	(2)	(3)	(4)	(5)	(6)
	WTP dummy	WTP	Intention to quit	Intention to avoid	Depth of processing	Risk perception
Nervous	-2.29 (.06)***	-.03 (.002)***	1.78 (.03)***	2.06 (.03)***	1.44 (.02)***	1.87 (.02)***
Const		.03 (.00)***	-.67 (.09)***	-.36 (.08)***	.53 (.05)***	-.20 (.05)***
N. Obs	15324	21768	20713	35207	55915	55920
LR test	1265.47***					
F test		154.07***	2161.24***	3958.36***	4038.62***	6067.76***
R2		.01	.05	.06	.04	.05
Hausman test	468.32***	60.92***	500.44***	882.38***	817.98***	1210.63***

Note: All the estimations are done with Two Stages Fixed Effects. Column (1) is a Fixed Effects Logit; columns (2)-(6) are Fixed Effects Linear regression. Each column corresponds to a regression; the dependent variable is indicated at the top of the column. Instruments are the 98 stimuli of the study (84 CWs and 14 textual warnings).

* indicates statistical significance at 10%

** at 5%

*** at 1%.

Standard errors in parenthesis. The Hausman test in all columns (including 1) is based on the linear estimator (Fixed Effects versus Two stages Fixed Effects).

**Table 6:** Effect of Anxiety.

	(1)	(2)	(3)	(4)	(5)	(6)
	WTP dummy	WTP	Intention to quit	Intention to avoid	Depth of processing	Risk perception
Anxious	-2.09	-.02 (.002)***	1.59 (.03)***	1.86 (.02)***	1.33 (.02)***	1.67 (.02)***
Const		.026 (.006)***	-.657 (.09)***	-.430 (.08)***	.427 (.05)***	-.209 (.05)***
N. Obs	15324	21768	20713	35207	55915	55920
LR test	1287.30***					
F test		144.94***	2123.26***	4015.54***	4267.26***	6012.33***
R2		.01	.06	.07	.05	.06
Hausman test	453.17***	79.09***	591.50***	1260.27***	1160.49***	1789.38***

Note: All the estimations are done with Two Stages Fixed Effects. Column (1) is a Fixed Effects Logit; columns (2)-(6) are Fixed Effects Linear regression. Each column corresponds to a regression; the dependent variable is indicated at the top of the column. Instruments are the 98 stimuli of the study (84 CWs and 14 textual warnings).

* indicates statistical significance at 10%

** at 5%

*** at 1%.

Standard errors in parenthesis. The Hausman test in all columns (including 1) is based on the linear estimator (Fixed Effects versus Two stages Fixed Effects).

**Table 7:** Effect of Fear.

	(1)	(2)	(3)	(4)	(5)	(6)
	WTP dummy	WTP	Intention to quit	Intention to avoid	Depth of processing	Risk perception
Fear	-.80 (.02)***	-.01 (.00)***	.61 (.01)***	.73 (.01)***	.50 (.00)***	.66 (.008)***
Const		-.00 (.00)	1.09 (.05)***	1.59 (.04)***	1.96 (.03)***	1.58 (.03)***
N. Obs	15324	21768	20713	35207	55915	55920
LR test	1261.84***					
F test		146.78***	2046.26***	3956.82***	3857.68***	6067.38***
R2		.01	.06	.07	.04	.06
Hausman test	147.88***	42.19***	128.21***	251.63***	120.87***	300.68***

Note: All the estimations are done with Two Stages Fixed Effects. Column (1) is a Fixed Effects Logit; columns (2)-(6) are Fixed Effects Linear regression. Each column corresponds to a regression; the dependent variable is indicated at the top of the column. Instruments are the 98 stimuli of the study (84 CWs and 14 textual warnings).

* indicates statistical significance at 10%

** at 5%

*** at 1%.

Standard errors in parenthesis. The Hausman test in all columns (including 1) is based on the linear estimator (Fixed Effects versus Two stages Fixed Effects).

**Table 8:** Effect of Disgust.

	(1)	(2)	(3)	(4)	(5)	(6)
	WTP dummy	WTP	Intention to quit	Intention to avoid	Depth of processing	Risk perception
Disgust	-.54 (.01)***	-.00 (.00)***	.42 (.009)***	.50 (.00)***	.32 (.00)***	.44 (.00)***
Const		-.01 (.00)***	2.17 (.03)***	2.86 (.03)***	2.88 (.02)***	2.77 (.02)***
N. Obs	15324	21768	20713	35207	55915	55920
LR test	1111.76***					
F test		159.77***	1849.11***	3585.00***	3185.65***	5248.40***
R2		.01	.05	.06	.03	.05
Hausman test	63.32***	32.71***	137.90***	254.38***	157.76***	327.56***

Note: All the estimations are done with Two Stages Fixed Effects. Column (1) is a Fixed Effects Logit; columns (2)-(6) are Fixed Effects Linear regression. Each column corresponds to a regression; the dependent variable is indicated at the top of the column. Instruments are the 98 stimuli of the study (84 CWs and 14 textual warnings).

* indicates statistical significance at 10%

** at 5%

*** at 1%.

Standard errors in parenthesis. The Hausman test in all columns (including 1) is based on the linear estimator (Fixed Effects versus Two stages Fixed Effects).

In all Tables, column (1) is a fixed effect logit regression and columns (2) through (6) are fixed effects linear regressions. Standard goodness of fit tests are reported in the last two rows. To make coefficients easier to interpret, as variables were elicited via different scales we report the effect size in terms of standard deviation from the mean for the dependent variable once we change the independent variable by one standard deviation from the mean. Here the standard deviation is the one within group, given that our coefficients are computed through fixed effects estimator. For the logit (column 2), we instead show the change in the odds ratio by a shift in the independent variable by one standard deviation, following standard practice. Thus, the effect of a one within group standard deviation (σ) of the independent variable over the odds of buying a cigarettes pack can be computed as the factor by which the latter changes, namely exp(*βσ*), where is the estimated coefficient. In the case of linear regression, the effect over the dependent variable can be expressed in terms of within group standard deviation of the dependent variable (σ_y)_ and is computed simply as *βσ/σ*_*y*_. In Table 9 we report the effect size together with mean and standard deviation of each variable.

**Table 9:** A summary of the size effects of emotional variables.

	(1)	(2)	(3)	(4)	(5)	(6)	(7)	(8)
	Mean	SD	WTP dummy	WTP	Intention to quit	Intention to avoid	Depth of processing	Risk perception
Valence	3.25	1.32	.21	.28	-.89	-.92	-.79	-.92
Arousal	5.43	1.73	.23	-.25	.86	.87	.74	.86
Distress	2.81	.89	.21	-.25	.86	.88	.79	.86
Shame	2.27	.77	.16	-.22	.93	1.2	.93	.93
Anger	2.45	.79	.16	-.34	1.11	1.11	.94	1.11
Anxiety	2.75	.84	.17	-.24	1.05	1.06	.92	1.05
Fear	4.25	1.55	.29	-.22	.74	.77	.64	.77
Disgust	3.68	1.70	.40	-.02	.56	.58	.45	.56
Mean			.39	-.05	3.72	4.71	4.10	4.40
SD			.22	.07	1.27	1.47	1.21	1.33

Note: Column (2) is the within subjects standard error. Columns (3)-(8) report the effect of one standard deviation change (around the mean) over the dependent variable. Column (3) is the effect over the odds ratio, while columns (4)-(8) are reported in terms of within group standard deviation of the dependent variable. The last two rows show mean and within group standard deviation of the dependent variable. The WTP is expressed in terms of percentage variation with respect to the average price in the country.

Our results are the following. A simple change of the emotional valence (i.e., from a positive to a negative affect) is extremely effective in dissuading decision makers from smoking. As reported in Table 9, exposition to a source of affect (i.e., a CW image) which decreases the valence from positive to negative by one standard deviation reduces the odds of buying by a factor of 80%, and the elicited price by 28% of a standard deviation. Similarly, a one standard deviation change towards negative valence increases intention to quit and avoid by around 90% of a standard deviation, while the impact is weaker on intention to talk to other people about the risk of tobacco (around 80% of one standard deviation).

As shown in Table 9, increasing emotional arousal by one standard deviation reduces the odds of buying a cigarette pack by 77% and reduces the WTP by one fourth of a standard deviation. Increasing arousal by the same amount increases conative and cognitive impact from about 75% of a standard deviation up to 87% of one standard deviation.

In general, we find that all basic emotions are very effective in dissuading smoking related cognitions and conation, as well as smoking behavior. Specifically we find (see Table 9; hereafter we use sd for standard deviation):

1. Increased distress (by one sd) reduces the odds of buying by 79% and the WTP by one fourth of a sd. The marginal effect on conative and cognitive variables is around 80–90% of a sd;
2. Increased shame (by one sd) reduces the odds of buying by 84% and the WTP by 22% of a sd. The marginal effect on conative and cognitive variables is around 93% of a sd for intention to quit and cognitive variable and is more than one sd in the case of the intention to avoid;
3. Increased anger (by one sd) reduces the odds of buying by 84% and the WTP by one third of a sd. The marginal effect on conative variables and risk perception is more than proportional (110% of one sd) and is 93% of a sd in the case of depth of processing;
4. Increased anxiety (by one sd) reduces the odds of buying by 83% and the WTP by 24% of a sd. The marginal effect on conative and cognitive variables is more than proportional (105% of a sd) except for the depth of processing, where it is around 92% of a sd;
5. Increased fear (by one sd) reduces the odds of buying by 71% and the WTP by 22% of a sd. The marginal effect on conative and cognitive variables ranges from 64% of a sd for the depth of processing variable up to 77% of a sd for the intention to avoid and the risk perception;
6. Increased disgust (by one sd) reduces the odds of buying by 60% and the WTP by 2% of a sd. The marginal effect on conative and cognitive variables ranges from 45% of a sd for the depth of processing variable to 0.58% of a sd for the intention to avoid variable.

In all cases the standard errors are very limited indicating low variability of the effect.

## Robustness

Before presenting our conclusions we discuss potential concerns about our results as well as some robustness analysis.

### 4.1 Concerns about the Broader Implications

One possible concern may originate from the fact that we document an important role of immediate strong emotions, but their effects may fade away, especially given the known difficulty of quitting or reducing smoking. From this standpoint, since smoking is an addiction, smoking cessation cannot be achieved effortlessly and pictorial warnings even with hard hitting strong emotions cannot be a quick fix.

Studies on smoking cessation show that one should follow participants over a period that ranges from 6 to 18 months to ascertain whether people who tried to quit actually succeeded in quitting ([56]; [57]; [58]). Indeed, from the addictive nature of smoking one may infer that the reaction to a given stimulus, although significant in the short run, will then vanish thereafter. People are very aware of the hazards of smoking and that leads them to change [41]. A large majority of smokers report that they want to quit but only between 2%-3% succeed every year ([59]; [41]). Even though this issue goes beyond the ambit of our study, it is worth discussing it in detail because it enables us to link the empirical claim about the role of immediate emotions to the macro level and indicates interesting areas for further research.

Recent quasi-experimental studies performed in Canada, where data are available for a decade now, suggest that pictorial warning have contributed to the reduction in smoking prevalence by almost 10% between 2001 and 2009 ([60]; [61]). In Brazil, where in the last 20 years smoking prevalence has gone down by 50%, a modeling simulation attributed to pictorial warnings (introduced also in 2001) at least 8% of this reduction [62].

The use of pictures to emphasize warnings on cigarette packaging is allegedly the most controversial among the recommendations contained in the 2003 WHO Framework Convention on Tobacco Control [63], p. 77). Analyses of tobacco industry documents suggest that in the face of tighter restrictions on conventional avenues for tobacco marketing the package represents an important marketing tool to create in-store presence and communicate brand image ([64]; [65]). The increasing release of limited edition novel packs has been shown to be an effective instrument to increase consumers’ appeal [66]. They provide an opportunity to communicate with smokers during the act of smoking ([67]; [65]). Whereas the industry did not oppose the first form of generic textual warnings, the companies have worked systematically at the international level to oppose more specific and stronger warnings and especially pictorial warnings [64]. The cigarette packs are a (cheap) form of portable advertisement, are prominently displayed in retail outlets and, evidently, once pictorial warnings are introduced they become an equally cheap way of counter-advertising and a threat to brand appeal. Actually, such strong opposition could be taken as further indirect confirmation that CWs are effective precisely because they curb the main channel used in advertisement to hit consumers’ emotions: branding and pack design. Virtually all smokers are exposed to such control measures, and pack-a-day smokers are potentially exposed to the warnings over 7000 times per year [68]. Non-smokers are also exposed to them in stores or whenever they are in contact with smokers handling the cigarettes pack.

Given that smoking is an addiction, pictorial warnings may not be a short-term panacea. However, the case of Canada and Brazil seem to suggest that over time they have an effect, and the research mandate is to attempt to explain the link between immediate strong emotions and lower prevalence. One intriguing hypothesis is that they contribute, as a sort of social marketing, to changing social norms—cultural prescriptions about what we ought and ought not to do through repeated immediate emotions, which then have multiplicative behavioral effects. Coherently with this hypothesis, there is an association between Tobacco industry advertisement presenting smoking as a form of female independence and sophistication and the changed social norms shown by the tremendous increase in smoking prevalence by women in the US. In fact, women in the US at the turn of the 20th century basically did not smoke and between 1940s and 1950s their smoking prevalence jumped [69]. The opposition by the industry to pictorial warnings is well in line with the hypothesis that the latter can act to reverse the pack channel and make it into an instrument to change social norms to the industry’s disadvantage. This line of argumentation hints at the need for theoretical and empirical development on the frontier of the micro-macro link that would shed light on the possible links between immediate emotions and socio-cultural norms.

### 4.2 Concerns about the type of Control conditions

A second possible concern is that by using text only (control conditions) and text-plus-image treatments, our estimates are restricted to the variations of emotional impulses and do not consider the emotional versus non-emotional content.

In fact, a Kruskal-Wallis test for emotional variables, using text only as range of variation, rejects the null hypothesis at 1% (results are shown in Table K in Section B of S1 File) and the estimates in the Section A of S1 File show that there is a clear effect of both CWs and TWs (Tables A-H of Section A of S1 File). As a result, there is exogenous variation in emotional scales among CWs, among TWs, and between CW and TW.

The answer to this objection can be threefold. First, it is of course very difficult to imagine some sort of control condition which is emotionally void: even a placebo treatment with a no warning pack would not be valid because some sort of anti-tobacco warning policy is currently in place everywhere in Europe. Second, a reasonable theoretical assumption is that going from zero to a strong emotional stimulus will indeed increase the effect, given that our estimates suggest that going from a positive amount to a strong emotional stimulus has already a sizable impact.

### 4.3 Concerns about the Experimental Methodology and Robustness

#### 4.3.1 Order Effects

A first methodological concern is that there may be a role of the order of exposition, due to the repeated measures design (every respondent is treated seven times). After running Kruskal-Wallis equality of population test for all the emotional variables, using the round of measurement as an independent variable, we can state that order of exposition matters. Results are reported in Table J in Section B of S1 File. In all cases the null hypothesis of equality is rejected at 1%. As a result we select the first two rounds of exposition for all the respondents (selection bias is not an issue due to the RCT design which guarantees counterbalancing) and re-run the estimation. In the vast majority of cases the new estimates include the old one in the confidence interval at 95%. In the case of intention to avoid and depth of processing the effect is—actually—*systematically higher* in the case of the new estimates. The only case in which the effect is lower is for the measure of valence, more precisely in the case of risk perception, but the difference is at the level of the second decimal digit. In conclusion, the order effect can be considered negligible as it does not change the main pattern of results.

#### 4.3.2 Country Effects

The experiments used in our study were run simultaneously in ten different countries. We now turn to investigate whether our results hold in each of the countries included. There are three reasons to expect some differences across countries. First, because in some countries CWs are already in use, while in others they are not: in the former group there could be some sort of decaying or ‘déjà vu’ effect with systematically weaker emotional reactions. Second, differences in culture and norms in general, and with regard to smoking in particular, may be reflected in systematic differences in the ways respondents judge their emotional reactions. Third, terminological nuances in the translation of the terms expressing very basic emotions may cause differences in the reported judgments.

Despite these possible differences, the assessment of the validity of our procedure suggests that our conclusions are very robust even controlling for potential differences by country, for the following reasons.

First, if we use random effect estimator to include country dummies in the specification, we can see that no change occurs in the direction and size of the relevant coefficients.

Second, if we allow for heterogeneous coefficients across countries, the results do not change qualitatively, maintaining the direction of change—the only measure for which we see a significant variation in the coefficients is the dummy for the willingness to buy, for a reduced number of emotional measures and only for Spain, but these results are not consistent with the other outcome variables—although there is a statistically significant difference in magnitude across countries (in particular, a chi-squared test with Bonferroni correction of equality of the coefficients across countries *rejects* the null hypothesis).

Third, our methodology removes all individual level variables that do not vary across exposition (fixed effects estimator). In other words, our estimators eliminates all differences across individuals in the outcome variables that may be due to culture, country, language, educational level and any other individual characteristics, which does not vary across expositions to the stimuli.

Finally, one should take into account that we aim at identifying the causal impact in reduced form at the European level. In other words, we are averaging across countries and culture to get a robust estimate of the effect at the European level. We are not aiming at a complete specification of the causal channel from emotions to behavior (which would be a structural estimation). In this complete causal specification, it is very likely that *culture matters*, and country differences could be informative for the specific policy implication at country level, but they are less relevant for our aim.

#### 4.3.3 Incentives

A third methodological concern is related to our use of non-incentivized behavioral measure (elicited willingness to buy and pay). Robust evidence suggests that for these kinds of tasks the lack of incentives introduces noise but not systematic differences in behavior [54]. In our study the use of repeated measure may actually increase even more the noise associated to the responses, but the estimates are very robust and with small standard errors. Moreover, the use of multiple sets of outcome variables (cognitive, conative, and behavioral) and emotional variables, all showing the same direction of change is further support for the thesis that an impact on actual behavior is very likely to occur.

#### 4.3.4 Social Desirability Bias

Finally, there may be an issue of social desirability bias, that is, the fact that responses may be affected by the desire of the respondents to give answers that would make them look good to, or please, the researchers. If this occurred, it would imply that somehow the respondents understand that this is a study related to anti-tobacco warnings and their ***self-reports*** would try to be socially desirable according to some social norms stating that some particular stimuli work better than others. They would respond more strongly to stimuli where a social norm might dictate stronger responses or they might guess that one type of response would support the study hypotheses more than other types of responses.

There is a vast theoretical and empirical literature on the emotional effects of pictures (for instance [37]; [38]; [39]). These contributions mainly rely on images from the International Affective Picture System (IAPS containing both Mild and Strong images) and recovering emotional reactions using the same SAM and PANAS scale we used. (Our stimuli are homogeneous with respect to the kind of picture contained in the IAPS with some stronger and some milder stimuli.) This literature posits and empirically shows three things: a) that when watching pictures individuals do not provide ***self-report*** but rather ***judgment*** of their emotional reactions; b) in various studies across nations, cultures, and social groups different types of pictures elicit different types of emotional reactions: strong images elicit more defensive (fear) or appetitive (pleasure) reactions than milder pictures; c) there is a very high correspondence between the judgment of emotions reported using SAM and PANAS and physiological measures of emotions such as skin-conductance, eye-blink, facial corrugator, etc. (i.e., [39]). It is difficult to imagine that results obtained on IAPS pictures across countries and culture are all affected by social desirability. Even less so given that scholars have found correspondence between reported judgments and physiological measures of emotional reactions.

Moreover, our design allows both detection and prevention of Social Desirability Bias.

A first argument against the presence of social desirability bias is the following. If we cut the sample at the first exposition, since the order is randomized we obtain a pure between-subjects design. This eliminates the social desirability effect because at the first exposition the respondent cannot perceive differences across stimuli having being exposed to just a single stimuli. We estimated the logit regression for the Willingness to Buy truncating the observation at the first exposition (there is no sample selection due to randomized order): the confidence intervals for the coefficients clearly confirm the results. More precisely, in the Logit regressions, the confidence interval for the Valence is [.15; .30], for the Arousal is [-.22; -.11], for Distress is [-.48; -.26], for Shame is [-.68; -.47], for Anger is [-.63; -.32], for Anxiety is [-.57; -.29], for fear is [-.22; -.11], for disgust is [-.16; -.07]. In other words, it is lower in magnitude but strongly significant and points in the same direction of the general conclusions indicated earlier. Thus the presence of Social Desirability Bias is highly unlikely.

Further, there is an argument against the presence of Social Desirability Bias based on the randomization and identification strategy. The randomization ensures all subjects are exposed to different types of stimuli (organ, social, and control) and our identification strategy, thus, uses the entire range of stimuli. This means that it considers both the differences across TWs and CWs, as well as between CWs and TWs. To the best of our knowledge, the current literature on risk perception does not suggest the presence of a social norm which is able to rank risks, for example, across typologies of cancers.

A third argument can be made, based on our experimental design. This is related to the presence of anti-tobacco content in all the warnings (as in all RCT examining anti-tobacco warnings, to the best of our knowledge). Let’s for a moment assume that subjects can really use social norms and guess the hypotheses and respond systematically to meet social norms and/or please the researcher. If this is the case they are likely to give both more emotional weight and less probability of buying to those stimuli that are high in terms of social desirability. Naturally then if we used stimuli unrelated to emotional reactions to tobacco, this would have facilitated the subjects in their social desirability/hypotheses guessing strategy. On the contrary, our strategy does not magnify the social desirability effects by providing a non-smoking related stimulus but rather minimizes it by providing many different smoking related stimuli and also several stimuli of the same type (six for a given type of risk). In fact, the literature on the emotional effect of pictures shows that “when emotional and non-emotional stimuli are presented simultaneously, emotional meaning captures initial overt orienting and engages attention early”, [38].

Finally, as stressed by the literature (e.g., see [70], [71]), social desirability effects are likely to be associated with some demographic variables, but our methodology (fixed effect estimator) removes, by construction, the effect of the demographic variables. As a result our estimated effect returns the average effect of emotions on behaviors once we control for the fact that non-smokers or other groups may be more or less responsive to anti-tobacco warnings.

Nevertheless, on this specific issue a detection strategy could be helpful. In fact, if social desirability effects occurred, it is more likely to exist in highly educated individuals, as suggested by the literature quoted above. Therefore, we ran Random Effects regressions of ‘willingness to buy’ introducing explicitly a dummy for tertiary education and an interaction effect between tertiary education and the emotional variables. The interaction effect is never significant: the coefficient of the interaction between the Valence and the tertiary education is -.00 (p = .91), for the Arousal is .01 (p = .84), for Distress is .05 (p = .60), for Shame is .12 (p = .43), for Anger is .05 (p = .72), for Anxiety is .04 (p = .74), for fear is .01 (p = .74), for disgust is .00 (p = .84). This evidence strongly suggests that education is not a channel through which emotions impact cognitions and behavior, and as such, these results provide further evidence against the relevance of SDB in our results.

## Concluding Remarks

Using data from a unique RCT study that was conducted simultaneously across 10 European countries with large sample sizes (N = 800 approx., per country), we attempted to isolate the effects of different emotions on smoking related cognitions, conation, and behavioral responses. A brief recapitulation of the findings is in order. First, we find that the basic emotions we examined in our study were successful in significantly reducing decision makers’ action propensity and thoughts related to smoking. Specifically, our findings suggest that the odds of buying a tobacco product can be reduced by 80% if the negative affect elicited by the images increases by one standard deviation. Additionally, the impact of different emotions (in terms of standard deviation change) on intentions to quit (smokers), intentions to avoid smoking (non-smokers), risk perceptions, and other cognitive outcomes ranges from half to more than one sd. Second, we also find that not all strong emotions behave alike. When the images elicited emotions like shame, anger, anxiety, and distress they were much more successful in reducing the decision maker’s odds of buying a tobacco product (on average by 82%) than when the images elicited emotions like fear and disgust (on average by about 66%). We would also like to reiterate that our estimation strategy eliminates the effect of fixed characteristics such as country, gender, and age, thus suggesting that the role of emotions goes beyond cultural and demographic stereotypes.

Our findings make several contributions both to the general literature on emotions in decision-making and to the more specific literature on health warnings (especially for tobacco consumption), and have important implications for the public policy domain.

1. We support the nascent literature showing that the impact of emotions on decision-making goes beyond cognition-mediated effects. Specifically we show that (a) immediate emotions can systematically alter behavioural responses, (b) strong emotions are particularly effective, and (c) different kinds of strong emotions vary in their efficacy; in other words, not all emotions are alike;
2. We contribute to the literature on designing nudges to improve health behaviors by (a) showing that in a domain like smoking behavior, one should look beyond the classical, effortless nudges based on inertia, the power of default behaviors, and other milder emotions (examples include competitions and light incentives to help people stop smoking [72]), and (b) suggest that since not all emotions are alike public policy decision makers should strive to design images that are heavier on shame, anger, anxiety, and distress (rather than simply on fear and disgust). This finding indicates that gruesome images, showing pictorial representation of body parts, children, and the dead may be an effective strategy to design combined warnings provided they elicit certain types of emotions.
3. By assessing the validity of our identification strategy, we contribute to the general literature on health warning and the specific literature in Tobacco studies on the value added of Combined Warnings. This becomes clear after looking at Tables A-H in Section A of S1 File, where an added value of CWs with respect to TWs emerges. In particular, we contribute to the literature on health warnings in general [73] by confirming (a) that imagery based information is more salient and elicits faster cognitive processing and stronger conative attitudes through more intense emotional reactions, and (b) that these effects occur without any sign of reactance. We also corroborate the tobacco specific literature comparing the relative efficacy of pictorial warnings to that of textual warning [74], showing that the former are more effective than the latter in shaping behavioural, cognitive, and conative reactions (i) with the largest RCT multi-country study that has higher external validity than most studies published so far (10 countries representing different cultural-institutional blocks), and (b) by using a more comprehensive set of response variables that better enable us to explore the mechanism through which pictorial warnings work.

At a time when in the US the courts have struck down the nine pictorial warnings proposed by the Federal Drug Administration (FDA) as part of the mandate received from the “Family Smoking Prevention and Tobacco Control Act (FSPTCA)” on the grounds of insufficient evidence of their capacity to achieve their declared goals ([75]; [76]; [77]; [78]), our study (a) provides very robust evidence of the fact that the Combined Warnings selected by the European Commission for implementing the Directives are effective in achieving the dissuasive goals and (b) shows the emotional mechanisms by which they have such effects on behavioural choices and on intentions. As argued, these CWs cannot be expected to be a quick fix for a recalcitrant addiction like smoking. However, the CWs are very economical ways to reduce the effectiveness of the marketing messages of the tobacco industry and to contribute to change in social norms and the ensuing behaviors in the middle term. In order to further corroborate this evidence by looking at actual and more distal outcomes (smoking cessation and overall smoking prevalence) quasi-experimental studies such as those conducted in Canada and Brazil (cited earlier) should be conducted in several European countries taking advantage of the fact that the actual implementation of the new directive (and of the corresponding CWs) will be staggered across time and space. This gives policy-makers ample time to design and deploy appropriate CWs as per the findings documented in our study.

## Supporting Information

S1 File Supplementary Online Materials. Includes: First Stage Regressions (Section A: Tables A-H), Robustness checks (Section B: Tables I-K), Full Experimental protocol (Section C), Experimental Flow (Section D), Examples of CW (Section E: Figure A) and TW (Section E: Figure B), Number of observations per treatment (Section F, Table L).
(DOCX)
