# Supplementary material for: Pathos & Ethos: Emotions and Willingness to Pay for Tobacco Products
Source: PLoS One. 2015 Oct 20;10(10):e0139542. doi: 10.1371/journal.pone.0139542 (PMC4618929; doi:10.1371/journal.pone.0139542)
Supplement: S1 File — Includes: First Stage Regressions (Section A: Tables A-H), Robustness checks (Section B: Tables I-K), Full Experimental protocol (Section C), Experimental Flow (Section D), Examples of CW (Section E: Figure A) and TW (Section E: Figure B), Number of observations per treatment (Section F, Table L). (DOCX) [file pone.0139542.s001.docx]

S1 File: Supplementary Online Materials

Section A: First Stage Regressions

We present the first stage regressions where the endogenous explanatory variables are regressed over the entire set of stimuli. The results are reported in eight separate Tables (Tables A-H), one per emotional variable. It is apparent that the emotional variation across stimuli is significant.

**Table A – First Stage Regression for the Valence Variable**

|  | **TM1** | **TM2** | **TM3** | **TM4** | **TM5** | **TM6** | **TM7** | **TM8** | **TM9** | **TM10** | **TM11** | **TM12** | **TM13** | **TM14** |
| --- | --- | --- | --- | --- | --- | --- | --- | --- | --- | --- | --- | --- | --- | --- |
| **CW1** | 2,36  (.07)*** | 3,13  (.08)*** | 3,98  (.08)*** | 3,18  (.08)*** | 3,77  (.07)*** | 3,42  (.07)*** | 2,20  (.07)*** | 2,89  (.08)*** | 4,13  (.07)*** | 3,89  (.07)*** | 2,83  (.07)*** | 3,02  (.07)*** | 3,28  (.07)*** | 2,99  (.07)*** |
| **CW2** | 2,41  (.07)*** | 3,22  (.07)*** | 3,4  (.07)*** | 3,42  (.08)*** | 2,71  (.08)*** | 2,86  (.07)*** | 2,64  (.07)*** | 2,58  (.08)*** | 4,16  (.08)*** | 3,90  (.07)*** | 3,52  (.07)*** | 2,90  (.07)*** | 3,16  (.07)*** | 3,05  (.07)*** |
| **CW3** | 2,15  (.08)*** | 3,15  (.08)*** | 3,77  (.08)*** | 2,88  (.07)*** | 2,97  (.08)*** | 3,10  (.08)*** | 3,18  (.08)*** | 2,39  (.08)*** | 3,48  (.07)*** | 3,60  (.06)*** | 3,28  (.07)*** | 2,92  (.07)*** | 3,32  (.07)*** | 3.03  (.07)*** |
| **CW4** | 2,04  (.07)*** | 3,25  (.07)*** | 3,93  (.08)*** | 2,69  (.07)*** | 3.03  (.08)*** | 3,26  (.08)*** | 3,15  (.08)*** | 2,36  (.08)*** | 3,96  (.07)*** | 3,68  (.07)*** | 2,90  (.07)*** | 2,50  (.06)*** | 3,06  (.07)*** | 4,43  (.07)*** |
| **CW5** | 1,99  (.08)*** | 3,10  (.08)*** | 3,86  (.08)*** | 2,80  (.08)*** | 2,76  (.08)*** | 2,90  (.08)*** | 2,61  (.07)*** | 2,45  (.08)*** | 4,02  (.07)*** | 3,78  (.07)*** | 3,01  (.06)*** | 2,47  (.07)*** | 3,18  (.07)*** | 2,94  (.07)*** |
| **CW6** | 2,37  (.07)*** | 2,59  (.07)*** | 3,31  (.08)*** | 2,79  (.08)*** | 3,40  (.08)*** | 2,95  (.08)*** | 3,60  (.07)*** | 2,87  (.08)*** | 4,01  (.06)*** | 3,89  (.07)*** | 2,61  (.07)*** | 2,59  (.06)*** | 2,99  (.07)*** | 4,88  (.07)*** |
| **TM** | 3,54  (.07)*** | 3,69  (.07)*** | 3,90  (.07)*** | 3,43  (.07)*** | 3,93  (.07)*** | 3,86  (.07)*** | 3,84  (.06)*** | 3,87  (.07)*** | 3,96  (.07)*** | 4,01  (.07)*** | 3,73  (.07)*** | 3,12  (.07)*** | 3,62  (.07)*** | 4,38  (.08)*** |

Note: OLS regression. Number of obs. = 55923. F = 1844,46. R2 = 0,7640. CW stands for Combined Warning, TW for Text Warning. TM is the text message of each block of treatments. See Appendix E for list of CWs and TWs. TM1 is Mouth and Throat, TM2 is Strokes and disability, TM3 is blindness, TM4 is lung cancer, TM5 is Damages Lung, TM6 is Heart Attack, TM7 is Clogs Arteries, TM8 is Teeth and Gums, TM9 is Impotence, TM10 is Fertility, TM11 is Harms Your Family, TM12 is Unborn Child, TM13 is Children Start Smoking, TM14 is Quit Smoking. ^*^ indicates statistical significance at 10%, ^**^ at 5% and ^***^ at 1%. Standard errors in parenthesis.

**Table B – First Stage Regression for the Arousal Variable**

|  | **TM1** | **TM2** | **TM3** | **TM4** | **TM5** | **TM6** | **TM7** | **TM8** | **TM9** | **TM10** | **TM11** | **TM12** | **TM13** | **TM14** |
| --- | --- | --- | --- | --- | --- | --- | --- | --- | --- | --- | --- | --- | --- | --- |
| **CW1** | 6,72  (.10)*** | 5,54  (.11)*** | 4,43  (.10)*** | 5,62  (.11)*** | 4,75  (.10)*** | 5,15  (.10)*** | 7,01  (.09)*** | 5,86  (.11)*** | 4,54  (.09)*** | 4,35  (.10)*** | 6,08  (.09)*** | 5,66  (.09)*** | 5,46  (.09)*** | 5,78  (.09)*** |
| **CW2** | 6,54  (.10)*** | 5,41  (.11)*** | 4,81  (.11)*** | 5,36  (.11)*** | 6,24  (.10)*** | 5,90  (.10)*** | 6,41  (.10)*** | 6,43  (.10)*** | 4,42  (.09)*** | 4,43  (.09)*** | 5,17  (.09)*** | 5,89  (.09)*** | 5,50  (.09)*** | 5,56  (.09)*** |
| **CW3** | 7,15  (.10)*** | 5,54  (.11)*** | 4,66  (.11)*** | 5,81  (.10)*** | 6,07  (.11)*** | 5,90  (.10)*** | 5,55  (.11)*** | 6,74  (.10)*** | 5,18  (.09)*** | 4,75  (.09)*** | 5,33  (.09)*** | 5,98  (.09)*** | 5,28  (.09)*** | 5,89  (.09)*** |
| **CW4** | 7,27  (.10)*** | 5,34  (.10)*** | 4,40  (.11)*** | 6,31  (.10)*** | 5,92  (.11)*** | 5,39  (.11)*** | 5,67  (.10)*** | 6,73  (.10)*** | 4,42  (.09***) | 4,81  (.09)*** | 5,80  (.09)*** | 6,36  (.09)*** | 5,81  (.10)*** | 4,30  (.09)*** |
| **CW5** | 7,55  (.09)*** | 5,42  (.11)*** | 4,64  (.11)*** | 6,01  (.10)*** | 6,09  (.11)*** | 5,85  (.10)*** | 6,39  (.10)*** | 6,70  (.10)*** | 4,27  (.09)*** | 4,56  (.09)*** | 5,54  (.09)*** | 6,46  (.09)*** | 5,52  (.09)*** | 5,99  (.09)*** |
| **CW6** | 6,78  (.10)*** | 6,33  (.10)*** | 5,37  (.10)*** | 6,08  (.11)*** | 5,10  (.11)*** | 5,70  (.11)*** | 4,85  (.11)*** | 5,88  (.11)*** | 4,39  (.09)*** | 4,26  (.09)*** | 6,39  (.09)*** | 6,15  (.09)*** | 5,74  (.10)*** | 3,89  (.09)*** |
| **TM** | 4,81  (.11)*** | 4,57  (.10)*** | 4,03  (.10)*** | 5,15  (.10)*** | 4,56  (.10)*** | 4,60  (.10)*** | 4,38  (.10)*** | 4,48  (.10)*** | 4,25  (.11)*** | 4,20  (.09)*** | 4,79  (.10)*** | 5,52  (.11)*** | 4,76  (.10)*** | 4,50  (.10)*** |

Note: OLS regression. Number of obs. = 55923. F = 2862,06. R2 = 0,8316. CW stands for Combined Warning, TW for Text Warning. TM is the text message of each block of treatments. See Appendix E for list of CWs and TWs. TM1 is Mouth and Throat, TM2 is Strokes and disability, TM3 is blindness, TM4 is lung cancer, TM5 is Damages Lung, TM6 is Heart Attack, TM7 is Clogs Arteries, TM8 is Teeth and Gums, TM9 is Impotence, TM10 is Fertility, TM11 is Harms Your Family, TM12 is Unborn Child, TM13 is Children Start Smoking, TM14 is Quit Smoking. ^*^ indicates statistical significance at 10%, ^**^ at 5% and ^***^ at 1%. Standard errors in parenthesis.

**Table C – First Stage Regression for the Distress Variable**

|  | **TM1** | **TM2** | **TM3** | **TM4** | **TM5** | **TM6** | **TM7** | **TM8** | **TM9** | **TM10** | **TM11** | **TM12** | **TM13** | **TM14** |
| --- | --- | --- | --- | --- | --- | --- | --- | --- | --- | --- | --- | --- | --- | --- |
| **CW1** | 3,39  (.06)*** | 2,83  (.06)*** | 2,33  (.05)*** | 2,79  (.06)*** | 2,38  (.05)*** | 2,62  (.05)*** | 3,34  (.06)*** | 2,82  (.06)*** | 2,31  (.04)*** | 2,41  (.05)*** | 3,23  (.05)*** | 3,14  (.05)*** | 3,01  (.05)*** | 3,13  (.05)*** |
| **CW2** | 3,25  (.06)*** | 2,87  (.06)*** | 2,52  (.06)*** | 2,71  (.06)*** | 3,12  (.06)*** | 3,00  (.05)*** | 3,21  (.06)*** | 2,96  (.06)*** | 2,29  (.05)*** | 2,45  (.05)*** | 2,70  (.05)*** | 3,15  (.05)*** | 3,10  (.05)*** | 3,19  (.05)*** |
| **CW3** | 3,32  (.06)*** | 2,94  (.06)*** | 2,30  (.06)*** | 2,87  (.06)*** | 2,97  (.06)*** | 2,89  (.06)*** | 2,96  (.06)*** | 3,15  (.06)*** | 2,48  (.05)*** | 2,59  (.05)*** | 2,80  (.05)*** | 3,14  (.05)*** | 2,84  (.05)*** | 3,22  (.05)*** |
| **CW4** | 3,38  (.06)*** | 2,84  (.05)*** | 2,33  (.05)*** | 2,98  (.06)*** | 2,84  (.06)*** | 2,77  (.06)*** | 2,95  (.06)*** | 3,09  (.06)*** | 2,25  (.04)*** | 2,57  (.05)*** | 3,15  (.05)*** | 3,48  (.05)*** | 3,15  (.05)*** | 2,39  (.05)*** |
| **CW5** | 3,70  (.06)*** | 2,96  (.05)*** | 2,41  (.06)*** | 3,08  (.06)*** | 3,01  (.06)*** | 2,96  (.06)*** | 3,05  (.06)*** | 3,16  (.06)*** | 2,24  (.04)*** | 2,50  (.05)*** | 3,12  (.05)*** | 3,41  (.05)*** | 2,95  (.05)*** | 3,31  (.05)*** |
| **CW6** | 3,39  (.06)*** | 3,17  (.05)*** | 2,63  (.06)*** | 3,23  (.06)*** | 2,70  (.06)*** | 2,95  (.06)*** | 2,52  (.05)*** | 2,87  (.06)*** | 2,29  (.04)*** | 2,28  (.04)*** | 3,29  (.05)*** | 3,35  (.05)*** | 3,24  (.05)*** | 2,05  (.04)*** |
| **TM** | 2,38  (.05)*** | 2,43  (.05)*** | 2,23  (.05)*** | 2,58  (.05)*** | 2,26  (.05)*** | 2,33  (.05)*** | 2,25  (.05)*** | 2,23  (.05)*** | 2,22  (.05)*** | 2,25  (.05)*** | 2,38  (.05)*** | 2,85  (.05)*** | 2,69  (.05)*** | 2,38  (.05)*** |

Note: OLS regression. Number of obs. = 55918. F = 2450,30. R2 = 0,8129. CW stands for Combined Warning, TW for Text Warning. TM is the text message of each block of treatments. See Appendix E for list of CWs and TWs. TM1 is Mouth and Throat, TM2 is Strokes and disability, TM3 is blindness, TM4 is lung cancer, TM5 is Damages Lung, TM6 is Heart Attack, TM7 is Clogs Arteries, TM8 is Teeth and Gums, TM9 is Impotence, TM10 is Fertility, TM11 is Harms Your Family, TM12 is Unborn Child, TM13 is Children Start Smoking, TM14 is Quit Smoking. ^*^ indicates statistical significance at 10%, ^**^ at 5% and ^***^ at 1%. Standard errors in parenthesis.

**Table D – First Stage Regression for the Shame Variable**

|  | **TM1** | **TM2** | **TM3** | **TM4** | **TM5** | **TM6** | **TM7** | **TM8** | **TM9** | **TM10** | **TM11** | **TM12** | **TM13** | **TM14** |
| --- | --- | --- | --- | --- | --- | --- | --- | --- | --- | --- | --- | --- | --- | --- |
| **CW1** | 2,51  (.06)*** | 2,15  (.05)*** | 1,91  (.05)*** | 2,17  (.06)*** | 2,08  (.05)*** | 2,06  (.05)*** | 2,49  (.06)*** | 2,48  (.06)*** | 2,09  (.04)*** | 1,96  (.04)*** | 2,74  (.05)*** | 2,46  (.05)*** | 2,52  (.05)*** | 2,28  (.05)*** |
| **CW2** | 2,31  (.06)*** | 2,16  (.05)*** | 1,99  (.05)*** | 2,08  (.05)*** | 2,46  (.06)*** | 2,32  (.05)*** | 2,41  (.06)*** | 2,60  (.06)*** | 2,06  (.05)*** | 1,95  (.04)*** | 2,43  (.05)*** | 2,52  (.05)*** | 2,59  (.05)*** | 2,32  (.05)*** |
| **CW3** | 2,63  (.06)*** | 2,22  (.06)*** | 1,93  (.05)*** | 2,22  (.05)*** | 2,27  (.06)*** | 2,23  (.06)*** | 2,21  (.06)*** | 2,75  (.06)*** | 2,12  (.04)*** | 2,07  (.04)*** | 2,59  (.05)*** | 2,47  (.05)*** | 2,40  (.05)*** | 2,37  (.05)*** |
| **CW4** | 2,51  (.07)*** | 2,27  (.05)*** | 1,89  (.05)*** | 2,22  (.06)*** | 2,24  (.05)*** | 2,11  (.05)*** | 2,22  (.05)*** | 2,67  (.06)*** | 1,99  (.04)*** | 2,07  (.04)*** | 2,72  (.05)*** | 2,51  (.05)*** | 2,63  (.05)*** | 2,12  (.04)*** |
| **CW5** | 2,79  (.06)*** | 2,20  (.05)*** | 2,04  (.05)*** | 2,39  (.06)*** | 2,30  (.06)*** | 2,29  (.06)*** | 2,38  (.06)*** | 2,63  (.06)*** | 1,97  (.04)*** | 2,13  (.05)*** | 2,40  (.05)*** | 2,64  (.05)*** | 2,58  (.05)*** | 2,48  (.05)*** |
| **CW6** | 2,57  (.06)*** | 2,35  (.05)*** | 2,09  (.05)*** | 2,39  (.06)*** | 2,07  (.05)*** | 2,25  (.06)*** | 1,99  (.05)*** | 2,59  (.06)*** | 2,01  (.04)*** | 1,96  (.04)*** | 2,85  (.06)*** | 2,64  (.05)*** | 2,63  (.05)*** | 1,87  (.04)*** |
| **TM** | 2,04  (.05)*** | 1,91  (.04)*** | 1,86  (.04)*** | 2,06  (.05)*** | 1,91  (.04)*** | 1,95  (.04)*** | 1,86  (.04)*** | 1,98  (.05)*** | 2,01  (.05)*** | 1,89  (.04)*** | 2,19  (.05)*** | 2,34  (.05)*** | 2,31  (.05)*** | 2,05  (.05)*** |

Note: OLS regression. Number of obs. = 55918. F = 1634,47. R2 = 0,7432. CW stands for Combined Warning, TW for Text Warning. TM is the text message of each block of treatments. See Appendix E for list of CWs and TWs. TM1 is Mouth and Throat, TM2 is Strokes and disability, TM3 is blindness, TM4 is lung cancer, TM5 is Damages Lung, TM6 is Heart Attack, TM7 is Clogs Arteries, TM8 is Teeth and Gums, TM9 is Impotence, TM10 is Fertility, TM11 is Harms Your Family, TM12 is Unborn Child, TM13 is Children Start Smoking, TM14 is Quit Smoking. ^*^ indicates statistical significance at 10%, ^**^ at 5% and ^***^ at 1%. Standard errors in parenthesis.

**Table E – First Stage Regression for the Anger Variable**

|  | **TM1** | **TM2** | **TM3** | **TM4** | **TM5** | **TM6** | **TM7** | **TM8** | **TM9** | **TM10** | **TM11** | **TM12** | **TM13** | **TM14** |
| --- | --- | --- | --- | --- | --- | --- | --- | --- | --- | --- | --- | --- | --- | --- |
| **CW1** | 3,03  (.06)*** | 2,53  (.06)*** | 2,12  (.05)*** | 2,57  (.06)*** | 2,27  (.05)*** | 2,40  (.05)*** | 3,01  (.06)*** | 2,61  (.06)*** | 2,19  (.04)*** | 2,03  (.04)*** | 2,69  (.05)*** | 2,52  (.05)*** | 2,49  (.05)*** | 2,59  (.05)*** |
| **CW2** | 2,85  (.06)*** | 2,52  (.05)*** | 2,23  (.05)*** | 2,43  (.06)*** | 2,85  (.06)*** | 2,67  (.05)*** | 2,91  (.06)*** | 2,73  (.06)*** | 2,16  (.05)*** | 2,15  (.04)*** | 2,33  (.04)*** | 2,60  (.05)*** | 2,47  (.05)*** | 2,48  (.04)*** |
| **CW3** | 3,08  (.06)*** | 2,59  (.06)*** | 2,16  (.05)*** | 2,55  (.06)*** | 2,62  (.06)*** | 2,68  (.06)*** | 2,53  (.06)*** | 2,93  (.06)*** | 2,31  (.04)*** | 2,24  (.05)*** | 2,45  (.05)*** | 2,52  (.05)*** | 2,34  (.05)*** | 2,61  (.05)*** |
| **CW4** | 3.01  (.06)*** | 2,45  (.05)*** | 2.07  (.05)*** | 2,64  (.06)*** | 2,60  (.06)*** | 2,48  (.06)*** | 2,56  (.05)*** | 2,80  (.06)*** | 2,06  (.04)*** | 2,13  (.04)*** | 2,57  (.05)*** | 2,69  (.05)*** | 2,55  (.05)*** | 2,11  (.04)*** |
| **CW5** | 3,26  (.06)*** | 2,47  (.05)*** | 2,23  (.05)*** | 2,74  (.06)*** | 2,64  (.06)*** | 2,66  (.06)*** | 2,79  (.06)*** | 2,89  (.06)*** | 2,06  (.04)*** | 2,13  (.04)*** | 2,58  (.05)*** | 2,71  (.05)*** | 2,41  (.05)*** | 2,69  (.05)*** |
| **CW6** | 3,04  (.06)*** | 2,82  (.06)*** | 2,50  (.05)*** | 2,79  (.06)*** | 2,35  (.05)*** | 2,54  (.06)*** | 2,21  (.05)*** | 2,67  (.06)*** | 2,15  (.04)*** | 2,06  (.04)*** | 2,68  (.05)*** | 2,67  (.05)*** | 2,57  (.05)*** | 1,83  (.03)*** |
| **TM** | 2,29  (.05)*** | 2,21  (.05)*** | 2,08  (.05)*** | 2,31  (.05)*** | 2,09  (.04)*** | 2,22  (.05)*** | 2,10  (.04)*** | 2,09  (.05)*** | 2,13  (.05)*** | 2,04  (.04)*** | 2,10  (.04)*** | 2,35  (.05)*** | 2,25  (.05)*** | 2,05  (.04)*** |

Note: OLS regression. Number of obs. = 55918. F = 1959,39. R2 = 0,7767. CW stands for Combined Warning, TW for Text Warning. TM is the text message of each block of treatments. See Appendix E for list of CWs and TWs. TM1 is Mouth and Throat, TM2 is Strokes and disability, TM3 is blindness, TM4 is lung cancer, TM5 is Damages Lung, TM6 is Heart Attack, TM7 is Clogs Arteries, TM8 is Teeth and Gums, TM9 is Impotence, TM10 is Fertility, TM11 is Harms Your Family, TM12 is Unborn Child, TM13 is Children Start Smoking, TM14 is Quit Smoking. ^*^ indicates statistical significance at 10%, ^**^ at 5% and ^***^ at 1%. Standard errors in parenthesis.

**Table F – First Stage Regression for the Anxiety Variable**

|  | **TM1** | **TM2** | **TM3** | **TM4** | **TM5** | **TM6** | **TM7** | **TM8** | **TM9** | **TM10** | **TM11** | **TM12** | **TM13** | **TM14** |
| --- | --- | --- | --- | --- | --- | --- | --- | --- | --- | --- | --- | --- | --- | --- |
| **CW1** | 3,34  (.06)*** | 2,88  (.06)*** | 2,32  (.05)*** | 2,89  (.06)*** | 2,54  (.05)*** | 2,72  (.06)*** | 3,31  (.06)*** | 2,87  (.06)*** | 2,36  (.05)*** | 2,30  (.05)*** | 3,09  (.05)*** | 2,83  (.05)*** | 2,90  (.05)*** | 2,93  (.05)*** |
| **CW2** | 3,20  (.06)*** | 2,80  (.06)*** | 2,50  (.06)*** | 2,74  (.06)*** | 3,17  (.06)*** | 3,01  (.05)*** | 3,19  (.05)*** | 3,02  (.06)*** | 2,37  (.05)*** | 2,38  (.05)*** | 2,66  (.04)*** | 2,94  (.05)*** | 2,89  (.05)*** | 2,88  (.05)*** |
| **CW3** | 3,36  (.06)*** | 2,91  (.06)*** | 2,38  (.05)*** | 2,83  (.05)*** | 2,91  (.06)*** | 2,96  (.06)*** | 2,84  (.06)*** | 3,17  (.06)*** | 2,51  (.05)*** | 2,49  (.05)*** | 2,77  (.05)*** | 2,88  (.05)*** | 2,65  (.05)*** | 2,92  (.05)*** |
| **CW4** | 3,40  (.06)*** | 2,72  (.05)*** | 2,27  (.05)*** | 2,95  (.06)*** | 2,90  (.06)*** | 2,84  (.06)*** | 2,87  (.06)*** | 3,12  (.06)*** | 2,22  (.04)*** | 2,45  (.04)*** | 2,99  (.05)*** | 3,02  (.05)*** | 3,01  (.05)*** | 2,36  (.05)*** |
| **CW5** | 3,56  (.06)*** | 2,79  (.06)*** | 2,47  (.06)*** | 3,00  (.06)*** | 2,92  (.06)*** | 3,05  (.06)*** | 3,06  (.06)*** | 3,19  (.06)*** | 2,28  (.04)*** | 2,38  (.05)*** | 2,97  (.05)*** | 3,10  (.05)*** | 2,81  (.05)*** | 3,04  (.05)*** |
| **CW6** | 3,31  (.06)*** | 3,13  (.05)*** | 2,71  (.05)*** | 3,10  (.06)*** | 2,63  (.06)*** | 2,89  (.06)*** | 2,45  (.05)*** | 2,90  (.06)*** | 2,34  (.04)*** | 2,26  (.04)*** | 3,02  (.05)*** | 3,07  (.05)*** | 2,98  (.05)*** | 2,05  (.04)*** |
| **TM** | 2,55  (.05)*** | 2,56  (.05)*** | 2,33  (.05)*** | 2,63  (.05)*** | 2,35  (.05)*** | 2,50  (.05)*** | 2,39  (.05)*** | 2,34  (.05)*** | 2,33  (.05)*** | 2,28  (.05)*** | 2,43  (.05)*** | 2,75  (.06)*** | 2,58  (.05)*** | 2,33  (.05)*** |

Note: OLS regression. Number of obs. = 55918. F = 2347,21. R2 = 0,8061. CW stands for Combined Warning, TW for Text Warning. TM is the text message of each block of treatments. See Appendix E for list of CWs and TWs. TM1 is Mouth and Throat, TM2 is Strokes and disability, TM3 is blindness, TM4 is lung cancer, TM5 is Damages Lung, TM6 is Heart Attack, TM7 is Clogs Arteries, TM8 is Teeth and Gums, TM9 is Impotence, TM10 is Fertility, TM11 is Harms Your Family, TM12 is Unborn Child, TM13 is Children Start Smoking, TM14 is Quit Smoking. ^*^ indicates statistical significance at 10%, ^**^ at 5% and ^***^ at 1%. Standard errors in parenthesis.

**Table G – First Stage Regression for the Fear Variable**

|  | **TM1** | **TM2** | **TM3** | **TM4** | **TM5** | **TM6** | **TM7** | **TM8** | **TM9** | **TM10** | **TM11** | **TM12** | **TM13** | **TM14** |
| --- | --- | --- | --- | --- | --- | --- | --- | --- | --- | --- | --- | --- | --- | --- |
| **CW1** | 5,70  (.07)*** | 4,61  (.08)*** | 3,33  (.09)*** | 4,42  (.09)*** | 3,53  (.08)*** | 4,04  (.08)*** | 5,82  (.07)*** | 4,87  (.08)*** | 3,31  (.07)*** | 3,02  (.07)*** | 4,65  (.07)*** | 4,44  (.08)*** | 4,40  (.07)*** | 4,65  (.08)*** |
| **CW2** | 5,49  (.07)*** | 4,27  (.09)*** | 3,96  (.09)*** | 3,96  (.09)*** | 5,11  (.08)*** | 4,66  (.08)*** | 5,36  (.07)*** | 5,54  (.07)*** | 3,25  (.07)*** | 3,21  (.07)*** | 3,72  (.07)** | 4,42  (.08)*** | 4,23  (.07)*** | 4,39  (.07)*** |
| **CW3** | 5,91  (.07)*** | 4,63  (.08)*** | 3,60  (.09)*** | 4,73  (.08)*** | 4,86  (.08)*** | 4,73  (.08)*** | 4,60  (.09)*** | 5,73  (.07)*** | 4,00  (.08)*** | 3,46  (.08)*** | 3,74  (.07)*** | 4,61  (.07)*** | 3,84  (.08)*** | 4,60  (.07)*** |
| **CW4** | 6,01  (.07)*** | 4,25  (.08)*** | 3,30  (.08)*** | 5,04  (.08)*** | 4,62  (.09)*** | 4,23  (.09)*** | 4,64  (.08)*** | 5,50  (.08)*** | 3,12  (.07)*** | 3,74  (.07)*** | 4,44  (.08)*** | 5,13  (.07)*** | 4,50  (.08)*** | 2,75  (.07)*** |
| **CW5** | 6,30  (.05)*** | 4,41  (.08)*** | 3,35  (.09)*** | 4,93  (.08)*** | 4,93  (.09)*** | 4,85  (.08)*** | 5,29  (.07)*** | 5,63  (.07)*** | 2,99  (.07)*** | 3,20  (.07)*** | 4,34  (.07)*** | 5,20  (.07)*** | 4,14  (.08)*** | 4,71  (.07)*** |
| **CW6** | 5,56  (.07)*** | 5,18  (.08)*** | 4,39  (.08)*** | 4,78  (.08)*** | 3,92  (.09)*** | 4,68  (.09)*** | 3,93  (.09)*** | 4,95  (.08)*** | 3,02  (.07)*** | 3,10  (.07)*** | 4,80  (.08)*** | 4,74  (.08)*** | 4,64  (.08)*** | 2,18  (.06)*** |
| **TM** | 4,02  (.08)*** | 3,80  (.08)*** | 3,50  (.08)*** | 4,29  (.08)*** | 3,44  (.08)*** | 3,72  (.08)*** | 3,47  (.07)*** | 3,33  (.08)*** | 3,39  (.08)*** | 3,39  (.08)*** | 3,46  (.08)*** | 4,53  (.08)*** | 3,79  (.08)*** | 3,00  (.07)*** |

Note: OLS regression. Number of obs. = 55920. F = 2958,89. R2 = 0,8298. CW stands for Combined Warning, TW for Text Warning. TM is the text message of each block of treatments. See Appendix E for list of CWs and TWs. TM1 is Mouth and Throat, TM2 is Strokes and disability, TM3 is blindness, TM4 is lung cancer, TM5 is Damages Lung, TM6 is Heart Attack, TM7 is Clogs Arteries, TM8 is Teeth and Gums, TM9 is Impotence, TM10 is Fertility, TM11 is Harms Your Family, TM12 is Unborn Child, TM13 is Children Start Smoking, TM14 is Quit Smoking. ^*^ indicates statistical significance at 10%, ^**^ at 5% and ^***^ at 1%. Standard errors in parenthesis.

**Table H – First Stage Regression for the Disgust Variable**

|  | **TM1** | **TM2** | **TM3** | **TM4** | **TM5** | **TM6** | **TM7** | **TM8** | **TM9** | **TM10** | **TM11** | **TM12** | **TM13** | **TM14** |
| --- | --- | --- | --- | --- | --- | --- | --- | --- | --- | --- | --- | --- | --- | --- |
| **CW1** | 5,74  (.07)*** | 3,98  (.09)*** | 2,46  (.07)*** | 3,52  (.09)*** | 3,24  (.08)*** | 2,79  (.07)*** | 6,10  (.06)*** | 5,54  (.07)*** | 2,90  (.07)*** | 2,25  (.06)*** | 3,66  (.08)*** | 3,32  (.08)*** | 3,69  (.08)*** | 3,70  (.08)*** |
| **CW2** | 5,35  (.07)*** | 3,16  (.08)*** | 3,42  (.08)*** | 3,19  (.08)*** | 5,26  (.08)*** | 3,54  (.08)*** | 5,29  (.07)*** | 6,12  (.05)*** | 2,74  (.07)*** | 2,39  (.06)*** | 3,18  (.07)*** | 3,69  (.08)*** | 3,68  (.08)*** | 2,86  (.07)*** |
| **CW3** | 6,31  (.06)*** | 3,68  (.09)*** | 2,86  (.08)*** | 4,23  (.08)*** | 4,75  (.08)*** | 4,72  (.09)*** | 3,64  (.09)*** | 6,22  (.05)*** | 3,72  (.08)*** | 2,52  (.06)*** | 3,58  (.08)*** | 3,85  (.08)*** | 3,29  (.08)*** | 3,25  (.08)*** |
| **CW4** | 6,31  (.06)*** | 3,51  (.08)*** | 2,35  (.07)*** | 4,92  (.08)*** | 4,80  (.09)*** | 3,68  (.09)*** | 3,88  (.08)*** | 6,07  (.06)*** | 2,89  (.07)*** | 3,13  (.07)*** | 3,93  (.08)*** | 3,91  (.08)*** | 3,95  (.08)*** | 2,38  (.06)*** |
| **CW5** | 6,52  (.04)*** | 3,31  (.08)*** | 2,84  (.08)*** | 4,58  (.08)*** | 5,02  (.08)*** | 3,93  (.09)*** | 5,51  (.07)*** | 6,19  (.05)*** | 2,34  (.06)*** | 2,57  (.07)*** | 3,15  (.07)*** | 4,15  (.08)*** | 3,67  (.08)*** | 3,43  (.08)*** |
| **CW6** | 5,31  (.08)*** | 4,48  (.08)*** | 4,24  (.08)*** | 3,53  (.09)*** | 3,30  (.09)*** | 3,73  (.09)*** | 3,14  (.08)*** | 5,54  (.07)*** | 2,38  (.06)*** | 2,80  (.07)*** | 4,81  (.08)*** | 3,77  (.08)*** | 4,40  (.08)*** | 1,91  (.05)*** |
| **TM** | 3,12  (.08)*** | 2,61  (.07)*** | 2,48  (.07)*** | 2,95  (.08)*** | 2,58  (.07)*** | 2,64  (.07)*** | 2,65  (.07)*** | 2,88  (.08)*** | 2,59  (.07)*** | 2,54  (.07)*** | 2,56  (.07)*** | 3,27  (.08)*** | 2,77  (.07)*** | 2,17  (.06)*** |

Note: OLS regression. Number of obs. = 55920. F = 2810,34. R2 = 0,8017. CW stands for Combined Warning, TW for Text Warning. TM is the text message of each block of treatments. See Appendix E for list of CWs and TWs. TM1 is Mouth and Throat, TM2 is Strokes and disability, TM3 is blindness, TM4 is lung cancer, TM5 is Damages Lung, TM6 is Heart Attack, TM7 is Clogs Arteries, TM8 is Teeth and Gums, TM9 is Impotence, TM10 is Fertility, TM11 is Harms Your Family, TM12 is Unborn Child, TM13 is Children Start Smoking, TM14 is Quit Smoking. ^*^ indicates statistical significance at 10%, ^**^ at 5% and ^***^ at 1%. Standard errors in parenthesis.

**Section B: Robustness checks**

In the following Table I we report Kruskal-Wallis test for the main demographic variables, using the stimuli as independent variable. In this way we can test if socio-demographic characteristics are balanced across conditions. The null hypothesis of equality across experimental conditions is an indirect test of the validity of the randomization algorithm. As can be seen equality of the means can never be rejected at standard significance level. We report the test per order of exposition because independence of observation is an assumption.

Table I. Kruskal-Wallis chi-2 test of the balancing of the socio-demographic characteristics across experimental conditions, by round of exposition.

|  | Sex | Age | Marriage | Smoking Status | Education | Occupation | Country | Household size |
| --- | --- | --- | --- | --- | --- | --- | --- | --- |
| Round 1 | 96.79  (.48) | 87.59  (.74) | 95.60  (.52) | 103.63  (.30) | 99.24  (.41) | 87.90  (.73) | 86.86  (.76) | 92.32  (.61) |
| Round 2 | 82.77  (.48) | 87.52  (.34) | 106.82  (.04) | 66.82  (.90) | 72.12  (.79) | 85.32  (.40) | 96.28  (.15) | 55.10  (.99) |
| Round 3 | 95.39  (.16) | 90.29  (.27) | 105.13  (.05) | 81.62  (.52) | 84.96  (.41) | 102.46  (.07) | 103.90  (.06) | 70.17  (.84) |
| Round 4 | 93.12  (.59) | 78.92  (.90) | 107.80  (.22) | 84.58  (.81) | 101.13  (.36) | 88.60  (.71) | 114.61  (.10) | 76.13  (.94) |
| Round 5 | 76.64  (.67) | 84.53  (.43) | 76.59  (.67) | 105.78  (.04) | 61.15  (.96) | 90.40  (.27) | 82.59  (.49) | 98.36  (.11) |
| Round 6 | 87.46  (.34) | 100.20  (.09) | 80.98  (.54) | 87.35  (.35) | 70.17  (.84) | 69.00  (.86) | 84.83  (.42) | 71.39  (.81) |
| Round 7 | 81.70  (.86) | 120.66  (.05) | 109.54  (.18) | 95.58  (.52) | 110.69  (.16) | 62.76  (.99) | 92.09  (.62) | 64.77  (.99) |

Note: chi-2 with ties, p value in parenthesis.

In Table J below, we report Kruskal-Wallis equality of population test across order of exposition for the main emotional variables.

Table J. Kruskal-Wallis test of the emotional responses across order of exposition.

|  | Valence | Arousal | Upset | Ashamed | Nervous | Anxious | Fear | Disgust |
| --- | --- | --- | --- | --- | --- | --- | --- | --- |
| Chi-2 | 340.83 | 329.97 | 2447.67 | 2047.21 | 2216.60 | 1769.73 | 251.66 | 375.66 |
| p-value | .00 | .00 | .00 | .00 | .00 | .00 | .00 | .00 |

Finally, in Table K we report the Kruskal-Wallis test of the emotional response across text only warnings.

Table K. Kruskal-Wallis test of the emotional responses across text warning only.

|  | Valence | Arousal | Upset | Ashamed | Nervous | Anxious | Fear | Disgust |
| --- | --- | --- | --- | --- | --- | --- | --- | --- |
| Chi-2 | 203.24 | 157.75 | 139.29 | 87.24 | 36.55 | 70.96 | 280.25 | 149.35 |
| p-value | .00 | .00 | .00 | .00 | .00 | .00 | .00 | .00 |

**Section C: The full experimental protocol**

Q1.1 Please tell us some information about you. What is your gender?

- Female (1)
- Male (2)

Q1.2 How old are you?

Q1.3 What is your nationality?

- Please select below... (1)

Q1.4 What is the highest level of education you have completed?

- primary school (1)
- some secondary school (2)
- completed high school (3)
- undergraduate university (4)
- postgraduate university (5)

Q1.5 Please indicate your occupation:

- Management, professional, and related (1)
- Service (2)
- Sales and office (3)
- Farming, fishing, and forestry (4)
- Construction, extraction, and maintenance (5)
- Production, transportation, and material moving (6)
- Government (7)
- Retired (8)
- Unemployed (9)
- Other (10)
- Student (11)

Q1.6 I would describe myself as...

- A smoker (1)
- A social smoker/special occasions smoker (2)
- A non smoker (4)
- An ex smoker (5)

Q1.7 Are you married?

- Yes (1)
- No (2)

Q1.8 Excluding yourself, how many household members are living with you?

- 0 (1)
- 1 (2)
- 2 (3)
- 3 (4)
- 4 (5)
- 5 (6)
- 6 (7)
- 7 (8)
- 8 (9)
- 9 (10)
- 10 (11)
- 11 (12)
- 12 (13)
- 13 (14)
- 14 (15)
- 15 (16)
- 16 (17)
- 17 (18)
- 18 (19)
- 29 (20)
- 20 (21)

Q1.9 Among the household members currently living with you and excluding yourself, how many persons are less than 16 years old?

- 0 (1)
- 1 (2)
- 2 (3)
- 3 (4)
- 4 (5)
- 5 (6)
- 6 (7)
- 7 (8)
- 8 (9)
- 9 (10)
- 10 (11)
- 11 (12)
- 12 (13)
- 13 (14)
- 14 (15)
- 15 (16)
- 16 (17)
- 17 (18)
- 18 (19)
- 29 (20)
- 20 (21)

Q2.1 We will show you seven different images that appear on cigarette packages, as shown below. For each image, after viewing it, we will ask you several questions, plus two short additional tasks. It should not take you more than 25 minutes in total. Please remember there are no right or wrong answers, we are simply interested in your response. Please click on the button below to start viewing the first image.


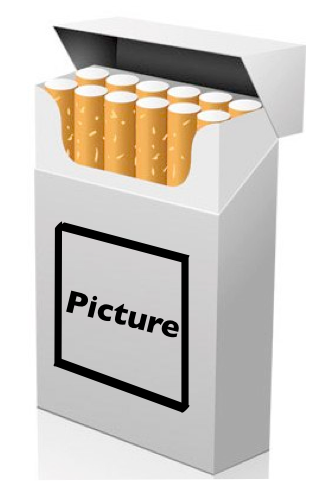


[All the questions below - until Q3.27 - are asked after each of the seven expositions, i.e. after each of the six CW and one text warning]

Q3.1 Please view the image below. After you have viewed this image to your satisfaction, please click on the button below to proceed with the rest of the survey, in which we will ask you several questions regarding this image.${lm://Field/1}

Q3.2 Timing

First Click (1)

Last Click (2)

Page Submit (3)

Click Count (4)

Q3.3 ${lm://Field/1} Please indicate on the scale below to what extent this image catches your attention.

______ 0 (1)

Q3.4 ${lm://Field/1} Thinking about this image, please select the figure below that most accurately shows how you feel.

- Image:SAM V1 (1)
- Image:SAM V2 (2)
- Image:SAM V3 (3)
- Image:SAM V4 (4)
- Image:SAM V5 (5)
- Image:SAM V6 (6)
- Image:SAM V7 (7)
- Image:SAM V8 (8)
- Image:SAM V9 (9)

Q3.5 Thinking about this image, please select the figure below that most accurately describes the intensity of your feelings.

- Image:SAM A1 (1)
- Image:SAM A2 (2)
- Image:SAM A3 (3)
- Image:SAM A4 (4)
- Image:SAM A5 (5)
- Image:SAM A6 (6)
- Image:SAM A7 (7)
- Image:SAM A8 (8)
- Image:SAM A9 (9)

Q3.6 Timing

First Click (1)

Last Click (2)

Page Submit (3)

Click Count (4)

Q3.7 ${lm://Field/1}Please indicate whether this image is disgusting.

______   (1)

Q3.8 Please indicate whether this image is frightening.

______   (1)

Q3.9 Timing

First Click (1)

Last Click (2)

Page Submit (3)

Click Count (4)

Answer If I would describe myself as... A smoker Is Selected Or I would describe myself as... A social smoker/special occasions smoker Is Selected

Q3.10 ${lm://Field/1}Please indicate on the scale below whether this image motivates you to quit.

______   (1)

Answer If I would describe myself as... A non smoker Is Selected Or I would describe myself as... An ex smoker Is Selected

Q3.11 ${lm://Field/1}Please indicate on the scale below whether this image motivates you to avoid smoking.

______   (1)

Q3.12 Please indicate whether this image makes you more concerned about the health risks of smoking.

______   (1)

Answer If I would describe myself as... A smoker Is Selected Or I would describe myself as... A social smoker/special occasions smoker Is Selected

Q3.13 Would this image make you want to cut back the number of cigarettes you smoke?

______   (1)

Q3.14 To what extent does this image make you think smoking is likely to cause mouth and throat cancer?

______   (1)

Q3.15 Timing

First Click (1)

Last Click (2)

Page Submit (3)

Click Count (4)

Q3.16 ${lm://Field/1}   This scale consists of a number of words that describe different feelings and emotions. Read each word and then mark the appropriate answer next to that word. Indicate to what extent you feel this way in reaction to this image. Use the following scale to record your answers.

______ Upset (1)

______ Ashamed (4)

______ Nervous (6)

______ Afraid (9)

______ Anxious (10)

Q3.17 Timing

First Click (1)

Last Click (2)

Page Submit (3)

Click Count (4)

Q3.18 ${lm://Field/1}Please indicate whether this image is repulsive.

______   (1)

Q3.19 Please indicate whether his image is scary.

______   (1)

Q3.20 Timing

First Click (1)

Last Click (2)

Page Submit (3)

Click Count (4)

Q3.21 ${lm://Field/1}   Please indicate whether you find this image personally relevant.

______   (1)

Q3.22 Please indicate whether you find this image believable.

______   (1)

Q3.23 Please indicate to what extent you think this image would encourage you to talk with other people about the dangers associated with smoking.

______   (1)

Answer If I would describe myself as... A non smoker Is Selected And Framing Condition Is Equal to 1

Q3.24 ${lm://Field/1}  Imagine that this image is on a cigarette pack like the one shown on the right. What is the maximum price at which you would consider buying it?           Prize

- £9,05 (1)
- £8,60 (2)
- £8,20 (3)
- £7,80 (4)
- £7,40 (5)
- £6,95 (6)
- £6,55 (7)
- £6,10 (8)
- £5,70 (9)
- £5,30 (10)
- £4,90 (11)
- I would not buy (12)

Answer If Framing Condition Is Equal to 2 And I would describe myself as... A non smoker Is Selected

Q3.25 ${lm://Field/1}   Imagine that this image is on a cigarette pack like the one shown on the right. What is the maximum price at which you would consider buying it?         Price

- £9,50 (1)
- £9,05 (2)
- £8,60 (3)
- £8,20 (4)
- £7,80 (5)
- £7,40 (6)
- £6,95 (7)
- £6,55 (8)
- £6,10 (9)
- £5,70 (10)
- £5,30 (11)
- £4,90 (12)
- I would not buy (13)

Answer If I would describe myself as... A smoker Is Selected And Framing Condition Is Equal to 1 Or I would describe myself as... A social smoker/special occasions smoker Is Selected And Framing Condition Is Equal to 1 Or I would describe myself as... An ex smoker Is Selected And Framing Condition Is Equal to 1

Q3.26   ${lm://Field/1}   Imagine that this image is on a cigarette pack like the one shown on the right. What is the maximum price at which you would be willing to buy it?         Price

- £9,05 (1)
- £8,60 (2)
- £8,20 (3)
- £7,80 (4)
- £7,40 (5)
- £6,95 (6)
- £6,55 (7)
- £6,10 (8)
- £5,70 (9)
- £5,30 (10)
- £4,90 (11)
- I would not buy (12)

Answer If I would describe myself as... A smoker Is Selected And Framing Condition Is Equal to 2 Or I would describe myself as... A social smoker/special occasions smoker Is Selected And Framing Condition Is Equal to 2 Or I would describe myself as... An ex smoker Is Selected And Framing Condition Is Equal to 2

Q3.27 ${lm://Field/1}   Imagine that this image is on a cigarette pack like the one shown on the right. What is the maximum price at which you would be willing to buy it?         Price

- £9,50 (1)
- £9,05 (2)
- £8,60 (3)
- £8,20 (4)
- £7,80 (5)
- £7,40 (6)
- £6,95 (7)
- £6,55 (8)
- £6,10 (9)
- £5,70 (10)
- £5,30 (11)
- £4,90 (12)
- I would not buy (13)

Q20.1 We will now show you some images that appear on cigarette packs. Most of these images were not shown to you earlier. On the next screen, we will (a) display the images and (b) request you to rank these images in terms of their ability to put you off smoking.

Q20.2 Please rank the following images, from 1 to 6, in terms of how effective you think they are in discouraging smoking. Rank the most effective by putting the number 1 in the text box, rank the second most effective by putting 2 in the text box, and so on until you have ranked all six images. Please note that the images are displayed randomly and the order does not represent how effective they are, so please rank them in the order that you think is most appropriate.

______   (1)

______   (2)

______   (3)

______   (4)

______   (5)

______   (6)

Answer If I would describe myself as... A smoker Is Selected

Q21.1 How soon after waking up do you usually smoke your first cigarette of the day?

- Within 5 minutes (1)
- 6-30 minutes (2)
- 31-60 minutes (4)
- After 60 minutes (6)

Answer If I would describe myself as... A social smoker/special occasions smoker Is Selected

Q21.2 How soon after waking up do you usually smoke your first cigarette of the day?

- Within 5 minutes (1)
- 6-30 minutes (2)
- 31-60 minutes (4)
- After 60 minutes (6)
- I do not smoke every day (7)

Answer If I would describe myself as... A smoker Is Selected Or I would describe myself as... A social smoker/special occasions smoker Is Selected Or I would describe myself as... An ex smoker Is Selected

Q21.3 How easy or difficult do you find to go without smoking for a whole day?

- Very Easy (1)
- Fairly Easy (2)
- Fairly Difficult (3)
- Very Difficult (4)

Answer If I would describe myself as... A smoker Is Selected Or I would describe myself as... A social smoker/special occasions smoker Is Selected

Q21.4 How many cigarettes do you usually smoke each day?

- 10 or less (1)
- 11-20 (2)
- 21-30 (3)
- 31 or more (7)

Answer If I would describe myself as... An ex smoker Is Selected

Q21.5 How many cigarettes did you used to smoke each day?

- 10 or less (1)
- 11-20 (2)
- 21-30 (3)
- 31 or more (7)

Answer If I would describe myself as... A smoker Is Selected Or I would describe myself as... A social smoker/special occasions smoker Is Selected

Q21.6 Overall, how confident are you that you can stop smoking altogether right now?

- Not At All Confident (1)
- Slightly Confident (2)
- Somewhat Confident (3)
- Very Confident (4)
- Completely Confident (5)

Answer If I would describe myself as... An ex smoker Is Selected

Q21.7 Overall, how confident are you that you will continue to resist to smoke altogether right now?

- Not At All Confident (1)
- Slightly Confident (2)
- Somewhat Confident (3)
- Very Confident (4)
- Completely Confident (5)

Answer If I would describe myself as... A non smoker Is Selected

Q21.8 Overall, how confident are you that you will continue not to smoke altogether right now?

- Not At All Confident (1)
- Slightly Confident (2)
- Somewhat Confident (3)
- Very Confident (4)
- Completely Confident (5)

Q22.1 What is your opinion about the questionnaire you just completed? We appreciate your comments.

**Section D: Experimental Flow**

Invitation sent to the Panel participant

Following quotas

Exclusion criteria:

Younger than 18 and older than 65 (n=0)

Refused to participate (n=2344)

Randomization n=8000

Condition 1 (n=1332)

Evaluate 3 Organ-Type Combined Warning from 3 different warning messages

Evaluate 3 Social-Type Combined Warning from 3 different warning messages

Evaluate one Textual Warning

Condition 2: (n=1338)

Evaluate 3 Organ-Type Combined Warning from 3 different warning messages

Evaluate one Textual Warning

Evaluate 3 Social-Type Combined Warning from 3 different warning messages

Condition 3 (n=1346)

Evaluate 3 Social-Type Combined Warning from 3 different warning messages

Evaluate 3 Organ-Type Combined Warning from 3 different warning messages

Evaluate one Textual Warning

Condition 4 (n=1329)

Evaluate 3 Social-Type Combined Warning from 3 different warning messages

Evaluate one Textual Warning

Evaluate 3 Organ-Type Combined Warning from 3 different warning messages

Condition 5 (n=1311)

Evaluate one Textual Warning

Evaluate 3 Organ-Type Combined Warning from 3 different warning messages

Evaluate 3 Social-Type Combined Warning from 3 different warning messages

Condition 6 (n=1344)

Evaluate one Textual Warning

Evaluate 3 Social-Type Combined Warning from 3 different warning messages

Evaluate 3 Organ-Type Combined Warning from 3 different warning messages

Analysis n=8000

**Section E: Examples of CW and TW**

**Figure A. Example of Combined Warnings (CW)**

**
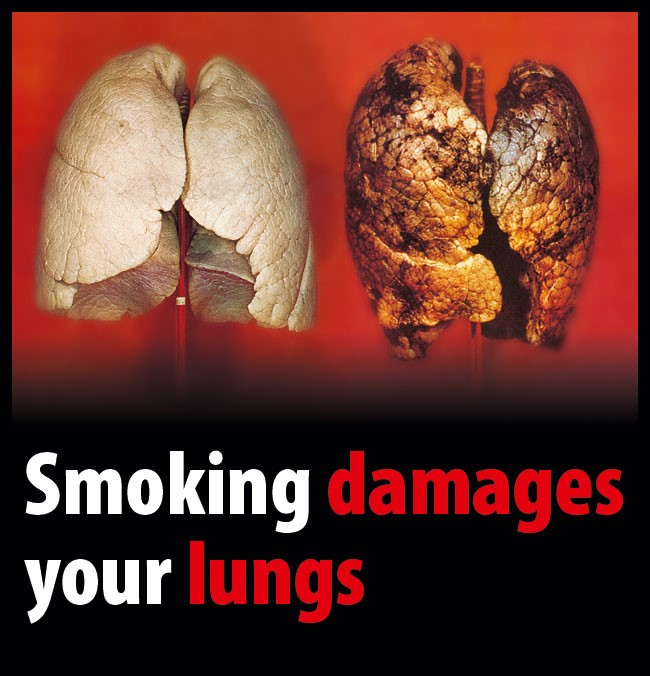
**

**Figure B. Example of Textual Warning (TW): Smoking clogs your arteries**

**
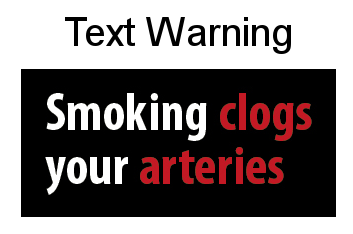
**

**Section F: Number of observations per treatment**

Table L. Number of observations per treatment

|  | TM1 | TM2 | TM3 | TM4 | TM5 | TM6 | TM7 | TM8 | TM9 | TM10 | TM11 | TM12 | TM13 | TM14 |
| --- | --- | --- | --- | --- | --- | --- | --- | --- | --- | --- | --- | --- | --- | --- |
| CW1 | 508 | 496 | 494 | 469 | 515 | 533 | 487 | 499 | 686 | 628 | 657 | 653 | 700 | 652 |
| CW2 | 531 | 492 | 496 | 496 | 504 | 502 | 513 | 514 | 616 | 673 | 688 | 687 | 680 | 697 |
| CW3 | 488 | 479 | 491 | 517 | 477 | 492 | 473 | 490 | 684 | 649 | 661 | 663 | 677 | 647 |
| CW4 | 488 | 542 | 472 | 511 | 516 | 475 | 506 | 496 | 670 | 703 | 656 | 672 | 628 | 635 |
| CW5 | 514 | 484 | 507 | 508 | 511 | 496 | 531 | 495 | 662 | 669 | 679 | 638 | 676 | 642 |
| CW6 | 468 | 513 | 519 | 500 | 472 | 487 | 507 | 490 | 687 | 670 | 650 | 684 | 656 | 698 |
| TW | 542 | 587 | 536 | 554 | 611 | 604 | 594 | 555 | 538 | 587 | 592 | 541 | 587 | 561 |

CW stands for Combined Warning, TW for Text Warning. TM is the text message of each block of treatments. See Appendix E for list of CWs and TWs. TM1 is Mouth and Throat, TM2 is Strokes and disability, TM3 is blindness, TM4 is lung cancer, TM5 is Damages Lung, TM6 is Heart Attack, TM7 is Clogs Arteries, TM8 is Teeth and Gums, TM9 is Impotence, TM10 is Fertility, TM11 is Harms Your Family, TM12 is Unborn Child, TM13 is Children Start Smoking, TM14 is Quit Smoking.
